# A Cu^I^Co^II^ cryptate for the visible light-driven reduction of CO_2_†

**DOI:** 10.1039/d3sc02679e

**Published:** 2023-10-27

**Authors:** Julia Jökel, Esma Birsen Boydas, Joël Wellauer, Oliver S. Wenger, Marc Robert, Michael Römelt, Ulf-Peter Apfel

**Affiliations:** a Fraunhofer UMSICHT Osterfelder Str. 3 46047 Oberhausen Germany ulf-peter.apfel@umsicht.fraunhofer.de ulf.apfel@rub.de; b Institute of Chemistry, Humboldt-Universität zu Berlin Brook-Taylor Str. 2 12489 Berlin Germany; c Department of Chemistry, Universität Basel St. Johanns-Ring 19 4056 Basel Switzerland; d Université Paris Cité, Laboratoire d'Electrochimie Moléculaire, CNRS F-75013 Paris France; e Institut Universitaire de France (IUF) F-76006 Paris France; f Inorganic Chemistry I, Ruhr-Universität Bochum Universitätsstr. 150 44801 Bochum Germany

## Abstract

Among the rare bimetallic complexes known for the reduction of CO_2_, Co^II^Co^II^ and Zn^II^Co^II^ hexamine cryptates are described as efficient photocatalysts. In close relation to the active sites of natural, CO_2_-reducing enzymes, we recently reported the asymmetric cryptand {N^S^N^N^}_m_ ({N^S^N^N^}_m_ = N[(CH_2_)_2_SCH_2_(*m*-C_6_H_4_)CH_2_NH(CH_2_)_2_]_3_N) comprising distinct sulphur- and nitrogen-rich binding sites and the corresponding Cu^I^M^II^ (M^II^ = Co^II^, Ni^II^, Cu^II^) complexes. To gain insight into the effect of metals in different oxidation states and sulphur-incorporation on the photocatalytic activity, we herein investigate the Cu^I^Co^II^ complex of {N^S^N^N^}_m_ as catalyst for the visible light-driven reduction of CO_2_. After 24 h irradiation with LED light of 450 nm, Cu^I^Co^II^-{N^S^N^N^}_m_ shows a high efficiency for the photocatalytic CO_2_-to-CO conversion with 9.22 μmol corresponding to a turnover number of 2305 and a high selectivity of 98% over the competing H_2_ production despite working in an acetonitrile/water (4 : 1) mixture. Experiments with mononuclear counterparts and computational studies show that the high activity can be attributed to synergistic catalysis between Cu and Co. Furthermore, it was shown that an increase of the metal distance results in the loss of synergistic effects and rather single-sited Co catalysis is observed.

## Abstract 2

A heterodinuclear Cu^I^Co^II^ complex of an asymmetric sulphur-containing cryptand gives rise to efficient visible light-driven CO_2_-to-CO conversion based on synergistic catalysis between Cu and Co.

## Graphical abstract

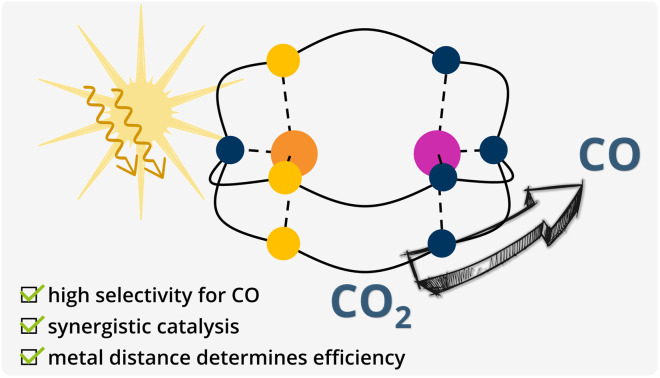

## Introduction

Artificial photosynthesis is a promising alternative to meet the increasing energy demand which has been, until now, mainly covered by the combustion of fossil fuels.^1–3^ With the use of abundant resources like sunlight and water, the reduction of CO_2_ into renewable fuels like CO, CH_4_, formic acid or methanol, is a viable approach for completing the carbon cycle.^4,5^ Although tremendous efforts have been devoted to the development of heterogeneous catalysts for the photocatalytic CO_2_ reduction, low yields together with a missing profound insight into reaction mechanisms make an industrial application not yet feasible.^6–8^

To get a fundamental understanding of important structural features or mechanistic details of an efficient photocatalytic CO_2_ reduction, homogeneous catalysis is a helpful tool.^9^ Moreover, the manifold spectroscopic techniques available for homogeneous catalysis allow for a specific fine-tuning of the catalyst's structure on a molecular level.^9–12^

Typically, homogeneous photocatalytic systems comprise three components: a photosensitiser for harvesting and converting light into electrochemical potential, a sacrificial reductant providing the required electrons and a molecular catalyst for accumulating and transferring them to the substrate.^13,14^ Due to the cruciality of the last step, many studies focus on the design of molecular catalysts.^11,15–17^

In contrast to the required harsh conditions and low selectivity associated with the artificial reduction of CO_2_, the naturally occurring enzymes CO dehydrogenases (CODHs) can convert CO_2_ selectively into CO under mild conditions.^18^ They commonly have heterobimetallic active sites comprised of frustrated Lewis pairs (*e.g.* Ni^0^Fe^II^, Cu^I^Mo^VI^) embedded in a protecting, sulphur-rich ligand environment.^18–21^

**Scheme 1:**
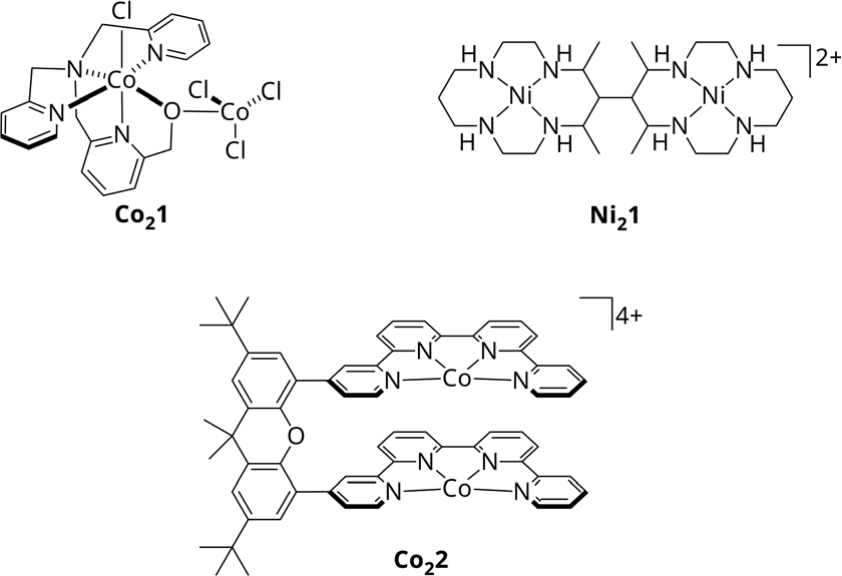
Molecular structures of Co_2_1, Ni_2_1 and Co_2_2.^26–28^

With CODHs being a role model, numerous homogeneous catalysts for the photocatalytic CO_2_ reduction have been developed in the past, though, most of them utilizing only one 3d metal within a macrocyclic chelating ligand.^15,16,22–25^ In addition, among the rare bimetallic catalysts like Co_2_1 or Ni_2_1 (Scheme 1), geometric constraints do often suppress synergistic catalysis between metal centres.^26,27^ One recent example for actual cooperative effects during CO_2_ reduction was described by Robert *et al.*^28^ Under visible light irradiation, the Co_2_ bi-quaterpyridine complex Co_2_2 (Scheme 1) selectively (max. 97%) reduces CO_2_ to formate in basic acetonitrile solution with a turnover frequency (TOF) of 0.23 min^−1^. Addition of a weak acid such as phenol shifts the reaction to CO with 99% selectivity and an increased catalytic rate of 13.8 min^−1^. Notably, the product outcome is dependent on the metal-bound η^2^ isomer of CO_2_, η^2^_O,O_ or η^2^_C,O_, stabilised under the respective conditions, leading to either formate or CO. However, in both cases, CO_2_ is bound between the Co centres, highlighting the potential of bimetallic complexes.

**Scheme 2:**
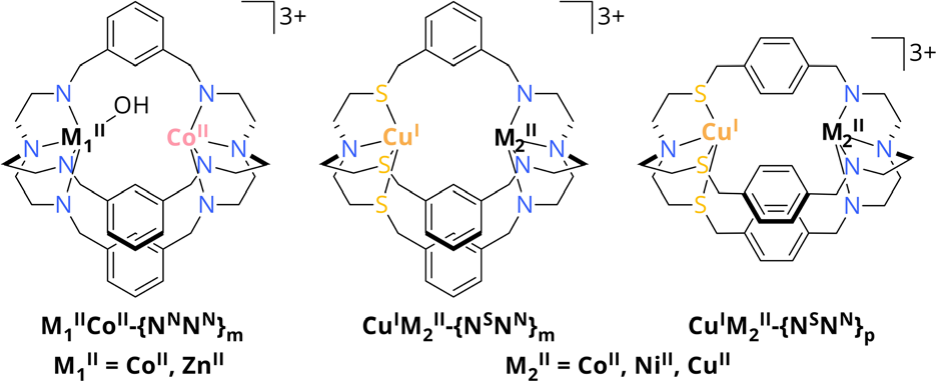
Schematic overview of Co^II^M_1_^II^-{N^N^N^N^}_m_ with M_1_^II^ = Co^II^, Zn^II^ and Cu^I^M_2_^II^-{N^S^N^N^}_m/p_ with M_2_^II^ = Co^II^, Ni^II^, Cu^II^. Hydrogen atoms are omitted for clarity.^32,33^

In the past, we amongst others reported on the ability of dinuclear azacryptates for the uptake of atmospheric CO_2_ as bicarbonate between the metal centres including Co_2_,^29^ Ni_2_,^30^ Cu_2_ ^31^ or Zn_2_ ^32^ complexes. Beyond these reports, Lu *et al.* reported on a dinuclear Co_2_ azacryptate (Co^II^Co^II^-{N^N^N^N^}_m_, Scheme 2) as photocatalyst for the visible light-driven CO_2_ conversion in a water-containing system with [Ru(phen)_3_](PF_6_)_2_ as photosensitiser (PS) and triethanolamine (TEOA) as sacrificial electron donor (SED). Due to synergistic effects between the Co^II^ centres, the cryptate was reported to reach a high catalytic rate of 0.47 s^−1^ compared to its mononuclear analogue with 0.04 s^−1^, while exhibiting a high selectivity of 98% for CO evolution even in the presence of water.^33^ The observed cooperativity was even more pronounced, when one Co^II^ was exchanged by Zn^II^ to yield Zn^II^Co^II^-{N^N^N^N^}_m_, resulting in an enhanced activity for CO_2_-to-CO conversion with 1.80 s^−1^ and a maintained high selectivity. Due to the stronger binding affinity of Zn^II^ over Co^II^ to OH^−^ in the critical C–O cleaving step, C–O bond cleavage is greatly promoted, highlighting the importance of understanding the role of metals in dual CO_2_ activation.^34^

These findings pointed our interest towards photocatalysts comprising azacryptates with not only metal centres of different kind but also oxidation states. Especially since the presence of a frustrated Lewis pair is crucial for the reduction of CO_2_ within the CODHs, we got interested in cooperative metal–metal effects for CO_2_ reduction.^18^ Along this line, we recently reported on the synthesis of the asymmetric cryptand {N^S^N^N^}_m_ which allows for the site-specific coordination of Cu^I^ and either Co^II^, Ni^II^ or Cu^II^ through sulphur incorporation within the ligand framework (Scheme 2). Furthermore, the distance between the metal centres can be increased when using {N^S^N^N^}_p_ as ligand.^35^

Thus, we herein report on the photocatalytic activity of Cu^I^Co^II^-{N^S^N^N^}_m_ towards CO_2_ reduction with additional focus on the influence of the metal distance. The selection of Cu^I^Co^II^-{N^S^N^N^}_m_ is based on the following considerations: (1) the Co^II^-{N^N^} site was already successfully reported for photocatalytic CO_2_ conversion^33,34^ and thus, allows for studying alterations of the other catalytic site. (2) Considering the active site's sulphur-rich environment in CO_2_-converting enzymes, using a catalyst with sulphur donors was desired. (3) Cu^I^-{N^S^} was synthetically easily accessible due to the matching donor–acceptor properties of Cu^I^ and sulphur and exhibits structural similarity to the active site of the [MoCu]-CODH.

## Results and discussion

In order to investigate the influence of mixed-valent metal centres on the overall CO_2_ reduction reactivity, we herein examined the stable heterobimetallic complex Cu^I^Co^II^-{N^S^N^N^}_m_ as potential photocatalyst under otherwise similar conditions as was reported by Lu *et al.*^33,35^ Like commonly described for bimetallic azacryptates,^31,33,34,36^Cu^I^Co^II^-{N^S^N^N^}_m_ readily absorbs CO_2_ which is indicated by a change of the UV/vis/NIR spectrum upon purging a MeCN/H_2_O (4 : 1) complex solution with CO_2_ for 5 min, indeed making it a promising candidate as a CO_2_ reduction catalyst. In the presence of CO_2_, the absorbance maxima at 490 nm and 618 nm decrease while the absorbance below 378 nm increases, consequently generating an isosbestic point (Fig. S1, ESI†) and thus, indicating the formation of a new species. The presence of a mass peak at *m*/*z* = 931.3 in the ESI-MS spectrum matching the [Cu^I^Co^II^-{N^S^N^N^}(HCO_3_)(ClO_4_)]^+^ fragment (Fig. S2B, ESI†) suggests a fixation of CO_2_ as bicarbonate. Moreover, the intensification of bicarbonate-related vibrations at 1641 cm^−1^ and 1449 cm^−1^ in the respective IR spectrum (Fig. S2A, ESI†) support this finding, albeit they coincide with ligand-associated vibrations.^31,36,37^ Notably, under Ar atmosphere, the UV/vis/NIR spectrum remained unchanged within 24 h and illustrates the high stability of Cu^I^Co^II^-{N^S^N^N^}_m_ in solution.

### Visible light-driven reduction of CO_2_ to CO with Cu^I^Co^II^-{N^S^N^N^}_m_ as catalyst

**Fig. 1:**
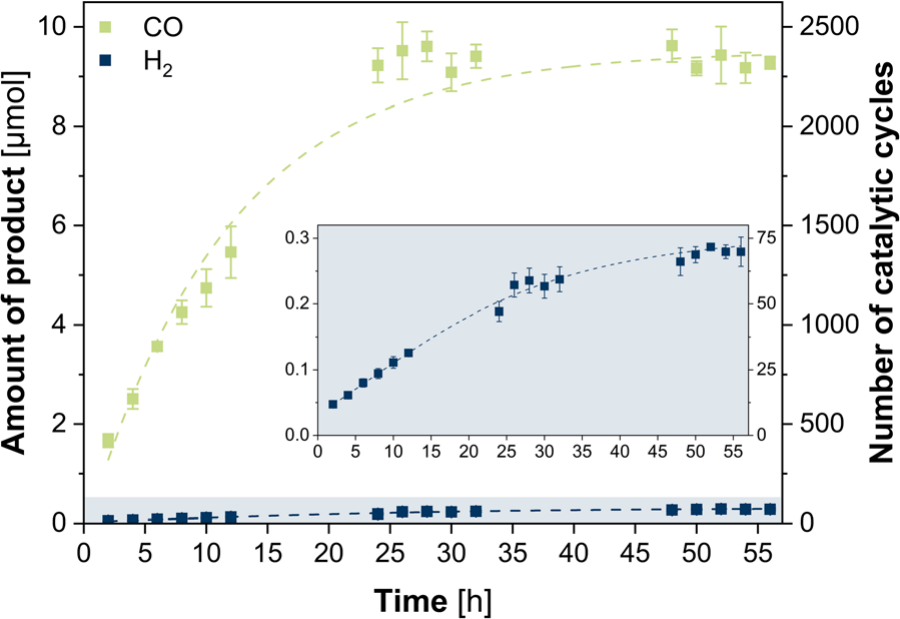
Photocatalytic evolution of CO (green) and H_2_ (blue) during 56 h catalysed by Cu^I^Co^II^-{N^S^N^N^}_m_ (2 μM) in a CO_2_-saturated MeCN/H_2_O (4 : 1) solution containing 0.4 mM [Ru(phen)_3_](PF_6_)_2_ and 0.3 M TEOA under irradiation with blue LED light (*λ* = 450 nm, 1200 mcd, irradiation area 0.8 cm^2^). Dashed lines represent the best fit of data.

The photocatalytic CO_2_ reduction experiments were performed using 2 μM Cu^I^Co^II^-{N^S^N^N^}_m_ as catalyst, 0.4 mM [Ru(phen)_3_](PF_6_)_2_ as photosensitizer and 0.3 M triethanolamine (TEOA) as sacrificial reductant in CO_2_-saturated MeCN/H_2_O (4 : 1) at room temperature. The photosystem was illuminated (450 nm LED, light intensity of 1200 mcd, irradiation area 0.8 cm^2^) for 56 h with two-hourly GC-BID analysis (except for hours 14–22 and 36–44) of the headspace composition as well as GC-MS analysis of the liquid phase composition after 56 h. All given values were averaged over three experiments with typical uncertainties of 2–8%. CO was observed as sole carbon-based product from CO_2_ reduction (Fig. S3 and S4, ESI†) with a steady increase of evolution during the first 24 h (Fig. 1). Beyond this point, the CO production ceased and the maximum value with 9.22 μM was reached, corresponding to a turnover number (TON) of 2305 and turnover frequency (TOF) of 1.60 min^−1^. Only H_2_ was formed as side product from proton reduction with 0.189 μmol (TON of 47, TOF of 3.28 × 10^−3^ min^−1^) after 24 h, leading to a high selectivity of 98% for CO_2_-to-CO conversion. Analysis of the liquid phase furthermore revealed the formation of acetaldehyde which can be attributed to the degradation of TEOA.^38^ The quantum yield was determined to be 0.15% using a potassium ferrioxalate actinometer (Fig. S5, ESI†). Contrary, in the absence of either Cu^I^Co^II^-{N^S^N^N^}_m_, [Ru(phen)_3_](PF_6_)_2_, TEOA or in the dark, no CO was detected, thus, showing the necessity of each component for the photocatalytic system (Fig. S6, ESI†). The small activity for H_2_ production with 8.85 × 10^−3^ min^−1^ (0.0510 μmol, TON of 13, 24 h) observed in experiments without Cu^I^Co^II^-{N^S^N^N^}_m_ is due to [Ru(phen)_3_](PF_6_)_2_ which can also act simultaneously as photosensitiser and catalyst for hydrogen evolution itself.^39,40^ Irradiation of an Ar purged solution instead of CO_2_ also generated no CO, excluding a potential CO production through degradation of any of the components of the photocatalytic system. Moreover, the presence of the proton source plays a pivotal role as well (Fig. S7, ESI†). While in neat MeCN the CO production rate is with 3.84 × 10^−2^ min^−1^ low (0.221 μmol, TON of 55, 24 h), addition of water with a ratio of MeCN/H_2_O 9 : 1 increased the activity to 1.08 min^−1^ (6.23 μmol CO, TON of 1558, 24 h). The best performance was achieved with the MeCN/H_2_O mixture of 4 : 1 while further increase of the water content to 1 : 1 limited the CO production to 1.37 × 10^−2^ min^−1^ (0.0791 μmol, TON of 20, 24 h). Isotope labelling experiments performed under a ^13^CO_2_ atmosphere followed by gas chromatography mass spectrometry analysis of the headspace, revealed the formation of ^13^CO (*m*/*z* = 29) as reduction product confirming that CO originates from CO_2_ (Fig. S8, ESI†).

Since the UV/vis/NIR spectrum of a MeCN/H_2_O (4 : 1) solution of 0.6 mM Cu^I^Co^II^-{N^S^N^N^}_m_ did not alter during 24 h irradiation, stability of the catalyst under irradiation can be anticipated (Fig. S1, ESI†) which is supported by the continuous production of CO during this time. HPLC analysis of the photocatalytic solution after 24 h irradiation followed by subsequent ESI-MS of the catalyst-containing fraction revealed a signal at *m*/*z* = 478.6 in the negative mode corresponding to [Cu^I^Co^II^-{N^S^N^N^}(BF_4_)(ClO_4_)(-3H)]^2−^ (Fig. S9, ESI†) and thus, further confirming the integrity of Cu^I^Co^II^-{N^S^N^N^}_m_. The durability of the photosystem, however, is limited to 24 h as shown by the ceasing CO production beyond this hour (Fig. 1). Since TEOA is present in large excess and the UV/vis/NIR spectrum of [Ru(phen)_3_](PF_6_)_2_ (Fig. S10, ESI†) shows a steady decrease of the absorbance maximum at 446 nm until 24 h, this finding can be attributed to the photodegradation of [Ru(phen)_3_](PF_6_)_2_. Furthermore, experiments in the presence of mercury revealed when considering the margins a similar CO production rate with 1.27 min^−1^ (7.34 μmol, TON of 1835, 24 h) and increased H_2_ production with 3.72 × 10^−2^ min^−1^ (0.214 μmol, TON of 54, 24 h) revealing that the catalytic reaction is predominantly homogeneous and does not result from elemental Cu or Co due to catalyst degradation. The absence of any particles in the photocatalytic solution after irradiation for 24 h is further confirmed by particle size analysis using laser diffraction (Fig. S11, ESI†). Consequently, reactivation experiments were performed in which fresh [Ru(phen)_3_](PF_6_)_2_ was added to the catalytic system after 26 h and 52 h (Fig. S12, ESI†). Notably, after addition of fresh photosensitiser, the CO production was increased again, achieving similar catalytic rates like after 24 h, with 1.70 min^−1^ (11.4 μmol, TON of 2850) and 1.48 min^−1^ (19.2 μmol, TON of 4800) after 2 h of the first and second re-addition, respectively, ultimately highlighting that [Ru(phen)_3_](PF_6_)_2_ is the main limiting factor herein.

### Synergistic catalysis and influence of metal distance on CO_2_ reduction

**Fig. 2:**
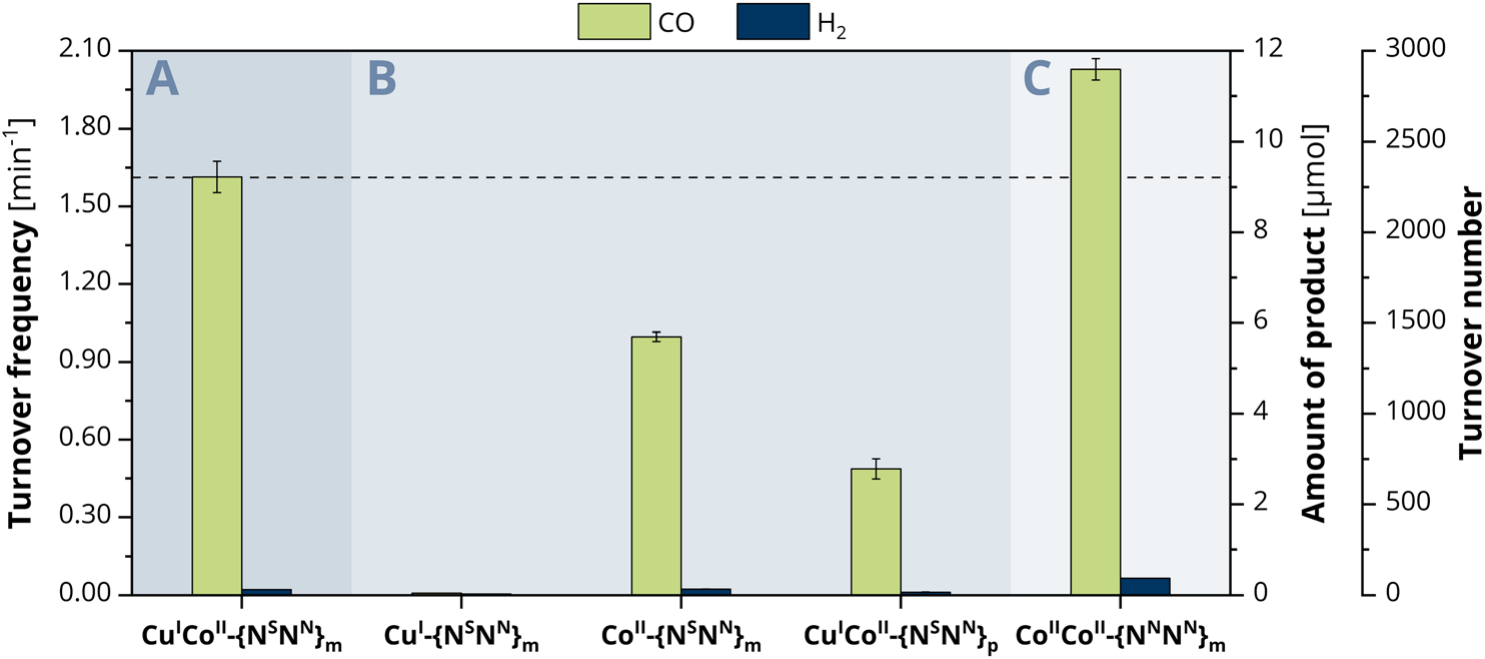
Photocatalytic evolution of CO (green) and H_2_ (blue) after 24 h irradiation with blue LED light (*λ* = 450 nm, 1200 mcd, irradiation area 0.8 cm^2^) of a CO_2_-saturated MeCN/H_2_O (4 : 1) solution containing 0.4 mM [Ru(phen)_3_](PF_6_)_2_ and 0.3 M TEOA with (A) Cu^I^Co^II^-{N^S^N^N^}_m_, (B) Cu^I^-{N^S^N^N^}_m_, Co^II^-{N^S^N^N^}_m_, and Cu^I^Co^II^-{N^S^N^N^}_p_ or (C) Co^II^Co^II^-{N^N^N^N^}_m_ as catalyst.

To explore the ongoing photocatalytic reaction in detail, investigations on the CO evolution kinetics were performed in a first step. Concentration-dependent experiments with 0.5, 1.0, 1.5 and 2 μM catalyst show at every hour a linear dependence of the concentration *versus* the amount of CO generated (Fig. S13, ESI†), suggesting a first-order reaction for the CO_2_-to-CO conversion. To consequently gain a more profound insight into the catalytic activity of the individual metals within Cu^I^Co^II^-{N^S^N^N^}_m_, we also employed the mononuclear counterparts Cu^I^-{N^S^N^N^}_m_ and Co^II^-{N^S^N^N^}_m_ as comparative catalyst systems (Fig. 2B).^35^ While the CO and H_2_ production with Cu^I^-{N^S^N^N^}_m_ was neglectable (CO: 0.01 min^−1^, 0.0464 μmol, TON of 12, H_2_: 4.86 × 10^−3^ min^−1^, 0.0279 μmol, TON of 7, 24 h), complex Co^II^-{N^S^N^N^}_m_ achieved a catalytic rate of 0.99 min^−1^ (5.70 μmol, TON 1425, 24 h) for CO and 2.34 × 10^−3^ min^−1^ (0.135 μmol, TON 34, 24 h) for H_2_, corresponding to a 98% selectivity of CO over H_2_ production. Thus, the nearly absent activity with Cu and the decreased activity with Co suggest that probably only the Co centre is the redox-active site during catalysis within Cu^I^Co^II^-{N^S^N^N^}_m_ but synergistically benefiting from the presence of the Cu centre.

We therefore became curious about whether the CO generation can be influenced by a variation of the metal distance between Cu^I^ and Co^II^. We previously described that the distance between the binding sites of the *para*-substituted analogue of the ligand, {N^S^N^N^}_p_, is roughly 1 Å larger than in {N^S^N^N^}_m_, giving a metal–metal distance of 6.578 Å within the related Cu^I^Cu^II^ cryptate.^35^ Due to the lack of a crystal structure of a corresponding *meta* cryptate, we consequently refer to the distance between the binding sites in the empty ligand and used Cu^I^Co^II^-{N^S^N^N^}_p_ as catalyst for comparison. Under otherwise identical conditions, Cu^I^Co^II^-{N^S^N^N^}_p_ achieved a CO production rate of 0.48 min^−1^ (2.78 μmol, TON of 695, 24 h) and a H_2_ production of 1.17 × 10^−2^ min^−1^ (0.0671 μmol, TON of 17, 24 h) (Fig. 2B). These values are significantly smaller compared to those observed with Cu^I^Co^II^-{N^S^N^N^}_m_ showing that the metal distance within Cu^I^Co^II^-{N^S^N^N^}_p_ is too large for synergistic catalysis to occur; therefore, it is possible that the Co^II^ site is solely responsible for the observed activity. Interestingly, Cu^I^Co^II^-{N^S^N^N^}_p_ has a smaller activity for CO production than the mononuclear catalyst Co^II^-{N^S^N^N^}_m_. We preliminarily attribute this not only to the altered metal distance but also lowered flexibility of the macrocycle caused by the *para*-substituted spacers bridging the two binding sites.

### Mechanistic studies of the CO_2_ reduction

#### Electrochemical investigations

The electrochemical properties of Cu^I^Co^II^-{N^S^N^N^}_m_ were determined by cyclic voltammetry (CV) in anhydrous MeCN using 0.1 M tetrabutylammonium hexafluorophosphate ([^*n*^Bu_4_N]PF_6_) as supporting electrolyte (Fig. S14A, ESI†). The CV reveals a redox couple at −0.88 V *vs.* Fc^+/0^ under inert conditions which is shifted anodically upon addition of CO_2_ to −0.84 V *vs.* Fc^+/0^. No significant increase in current was observed, indicating that in the absence of a proton source, no catalytic transformation of CO_2_ is going on. In MeCN/H_2_O (4 : 1), Cu^I^Co^II^-{N^S^N^N^}_m_ exhibits one primary reduction wave at −1.05 V *vs.* Fc^+/0^ and at −1.59 V *vs.* Fc^+/0^. Overlay with the CVs of the monometallic counterparts Cu^I^-{N^S^N^N^}_m_ and Co^II^-{N^S^N^N^}_m_ clarifies that the former wave can be assigned to the reduction of Co^II^ to Co^I^ and the latter one corresponds to the reduction of Cu^I^ (Fig. S14B, ESI†). The corresponding oxidations for Cu^0^ to Cu^I^ and Co^I^ to Co^II^ appear at −0.78 and −0.62 V *vs.* Fc^+/0^, respectively. Additionally, the herein measured potential window displays further the oxidation of Cu^I^ to Cu^II^ at −0.25 V followed by reduction at −0.76 V *vs.* Fc^+/0^. Upon saturation with CO_2_, the Co^II/I^ redox couple remains nearly unchanged in terms of current but with a minor anodic shift to −1.01 V. Furthermore, an additional diffusion-limited signal can be observed, suggesting an interaction with CO_2_, followed by a catalytic response at higher potentials (Fig. S14C and S15A, ESI†). Contrary, a pre-catalyst transformation was not observed in the linear sweep voltammogram (LSV) of Co^II^-{N^S^N^N^}_m_ in the presence of CO_2_ with a catalytic current increase from −1.44 V *vs.* Fc^+/0^, suggesting that it follows a different mechanism than Cu^I^Co^II^-{N^S^N^N^}_m_. This is also the case for Cu^I^-{N^S^N^N^}_m_ which additionally does not display a catalytic response at considerable potential, supporting its inactivity towards the photocatalytic CO_2_ reduction. Since the LSVs of Cu^I^Co^II^-{N^S^N^N^}_m_ and Cu^I^Co^II^-{N^S^N^N^}_p_ show a similar behaviour upon addition of CO_2_, no reason for the difference in reactivity within the photocatalytic CO evolution can be drawn from the electrochemical studies (Fig. S15A, ESI†).

#### Luminescence quenching

Stern–Volmer emission quenching experiments between the excited state of the photosensitiser [Ru(phen)_3_]^2+^* and either Cu^I^Co^II^-{N^S^N^N^}_m_ or TEOA revealed quenching rate constants of 4.13 × 10^7^ M^−1^ s^−1^ and 3.37 × 10^4^ M^−1^ s^−1^, respectively (Fig. S16 and S17, ESI†). Under experimentally relevant conditions, the reaction between triplet excited [Ru(phen)_3_]^2+^* and TEOA (*k*_TEOA_ = 1.01 × 10^4^ s^−1^) is roughly a factor of 120 faster than the reaction between [Ru(phen)_3_]^2+^* and Cu^I^Co^II^-{N^S^N^N^}_m_ (*k*_Cat_ = 82.6 s^−1^). Thus, electron transfer occurs firstly from TEOA to the excited photosensitiser and subsequently from [Ru(phen)_3_]^+^ to the catalyst. The electrochemical studies support that this electron transfer is thermodynamically accessible since the standard redox potential of [Ru(phen)_3_]^2+/+^ (−1.77 V *vs.* Fc^+/0^, Fig. S18, ESI†) is more negative than all redox couples determined for Cu^I^Co^II^-{N^S^N^N^}_m_. However, electron transfer to Co^II^ should be more likely than reduction of Cu^I^ due to its less cathodic reduction potential of −1.01 V compared to −1.54 V *vs.* Fc^+/0^, respectively.

#### Computational studies

**Scheme 3:**
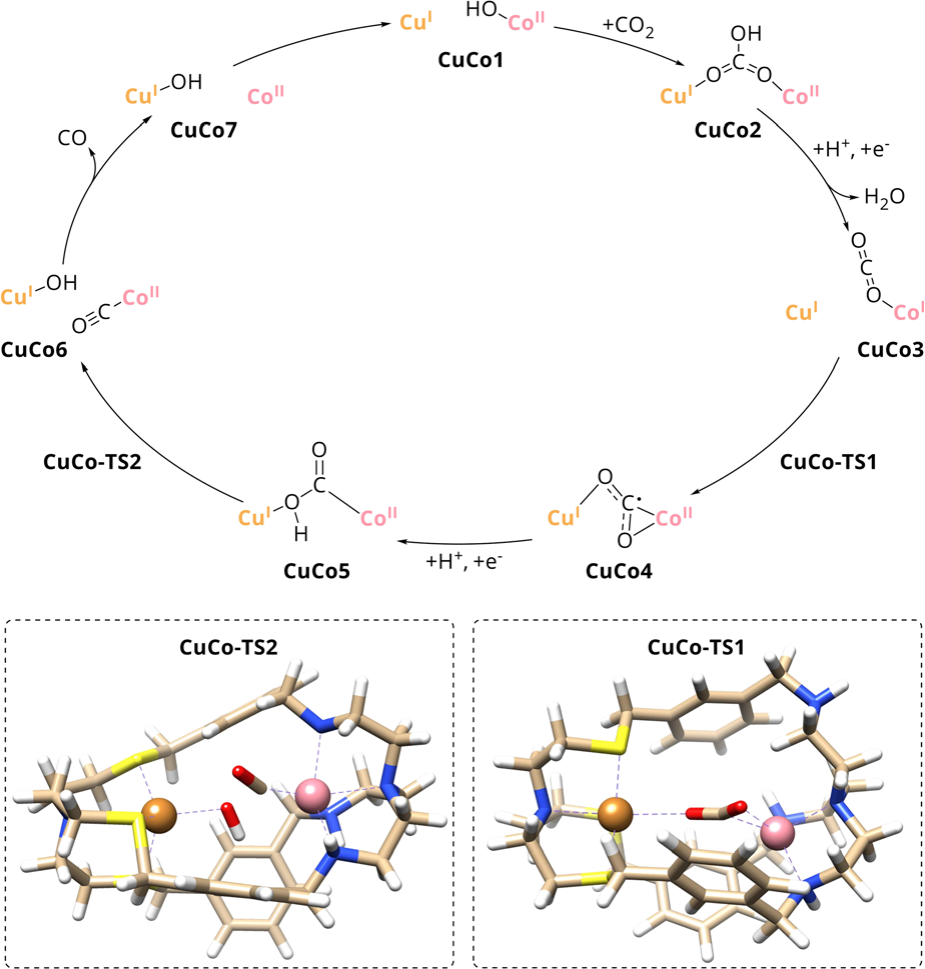
Proposed mechanism for CO_2_ reduction to CO with Cu^I^Co^II^-{N^S^N^N^}_m_ as catalyst. (Inset) Transition state structures for one electron CO_2_ reduction and C–OH bond cleavage, CuCo-TS1 and CuCo-TS2, respectively.

The synergistic catalysis between Cu^I^ and Co^II^ in the given coordination environments has been further investigated by computational calculations and a reaction pathway for the CO_2_-to-CO conversion by Cu^I^Co^II^-{N^S^N^N^}_m_ was proposed (Scheme 3 and Fig. S19, ESI†). Prior to any mechanistic study, the binding energies of potential axial ligands including CO_2_, MeCN, OH^−^ and CO with respect to ligation of the unreduced catalyst were computed and the hydroxyl anion was found to bind strongest. Since it is well known that the fixation of CO_2_ within bimetallic cryptates proceeds *via* hydroxo species as bicarbonate in basic solution, the hydroxylated catalyst CuCo1 that accommodates OH^−^ at the Co^II^-{N^N^} site is a reasonable starting point.^31–34,36,37^ Consequently, CO_2_ uptake results in the formation of the μ_2_,η^2^_O,O_ bicarbonate adduct CuCo2 with 6.9 kcal mol^−1^ reaction free energy, resulting from the cage-deformation upon binding of the ligand. This step is in good agreement with the aforementioned results from IR spectroscopy and mass spectrometry which suggested an incorporated bicarbonate ligand within Cu^I^Co^II^-{N^S^N^N^}_m_ in the presence of CO_2_. Subsequently, an exothermic proton-coupled electron transfer (PCET) accompanied by the release of water yields the Co^I^–CO_2_ complex CuCo3. Here, the spin density is entirely located at the Co^I^ centre and electron transfer to the lowest unoccupied molecular orbital (LUMO) of CO_2_ has not yet occurred which is reflected by its almost linear structure (∠ = 176.7°). Upon intramolecular electron transfer with a reaction free energy of 6.0 kcal mol^−1^ and energy barrier of 8.8 kcal mol^−1^, the CO_2_ radical anion is formed *via* transition state CuCo-TS1 which is accompanied by a reduction of the O–C–O angle and a change in binding mode from terminal to μ_2_,η^3^ (CuCo4). A further PCET yields the μ_2_,η^2^_C,O_ Co-COOH-Cu complex CuCo5 with the hydroxy moiety still weakly bound to the Cu centre. Subsequent C–OH bond cleavage *via* transition state CuCo-TS2 results in CuCo6 with Co-bound CO and Cu-bound OH^−^. Although a further reduction of the Co^II/I^–CO couple is favourable in terms of redox potential and binding energy of CO to cobalt (Tables S1 and S2, ESI†), the interaction between the two intracavitary ligands is rather unfavourable with a predicted energy barrier of 8.9 kcal mol^−1^. Thus, the following highly exothermic release of CO (−28.8 kcal mol^−1^) to yield CuCo7 together with the continuous mass transport of gaseous CO, is the driving force for this reaction step and preferred over a further reduction (+14.4 kcal mol^−1^). After migration of the hydroxyl group to the Co centre with an energy barrier of 7.1 kcal mol^−1^, the initial catalyst is restored, and the catalytic cycle can restart.

A comparison of the catalytic cycle with the experimental results of the mononuclear analogues reveals a good agreement of the predictions of the role of the individual metal within Cu^I^Co^II^-{N^S^N^N^}_m_. The nearly absent activity of Cu^I^-{N^S^N^N^}_m_ towards the reduction of CO_2_ is in line with Cu taking over an assistant role in binding during catalysis. In contrast, Co was experimentally proposed as redox-active site due to the activity of Co^II^-{N^S^N^N^}_m_ towards CO_2_-to-CO conversion which matches the role of Co during the catalytic cycle, too. To further strengthen these findings, smaller computed scaffold models comprising the specific tripodal ligand backbone with an open coordination site in axial position (Fig. S20, ESI†) were considered.^33^ While one-electron reduction of the Cu^I^-{N^S^} site alone entails a complex decomposition, addition of an electron to Cu^I^-{N^S^} and CO_2_ leads to the formation of the CO_2_˙^−^ that is only weakly bound to the catalyst (*d* = 2.83 Å). Hence, in accordance with the experimental results for Cu^I^-{N^S^N^N^}_m_, a pathway for the reaction between Cu^I^-{N^S^} and CO_2_ could not be established. Contrary to Cu^I^-{N^S^} and thus, in good agreement with the experimental results for Co^II^-{N^S^N^N^}_m_, a reaction profile for the Co^II^-{N^N^} site with CO_2_ was proposed by Lu *et al.*^33^ Therein, a simultaneous two-electron transfer step from the Co centre to CO_2_ yields a η^1^_C_ Co^III^–CO_2_^2−^ intermediate which is transformed in a PCET into Co^II^-COOH^−^. Subsequently, C–OH bond cleavage results in CO and OH^−^, both bound to Co, followed by release of the former. While the simultaneous two-electron reduction of CO_2_ in Co^II^-{N^N^} proceeds with an energy barrier of 19.5 kcal mol^−1^,^33^ the one for the stepwise reduction in Cu^I^Co^II^-{N^S^N^N^}_m_ with 10.4 kcal mol^−1^ is significantly lower which is which most likely due to the favourable interaction of Cu stabilising the CO_2_˙^−^ adduct in CuCo4. Furthermore, the energy barrier of the following C–OH bond cleavage is considerably lower for Cu^I^Co^II^-{N^S^N^N^}_m_ (8.9 kcal mol^−1^) compared to the reported value for the mononuclear case (18.1 kcal mol^−1^).^33^ Consequently, benefitting from the synergistic effect between the two metals, with Co^II^ as active site and Cu^I^ taking over an assistant role in binding, CO_2_ reduction to CO is more favoured in the dinuclear cryptate Cu^I^Co^II^-{N^S^N^N^}_m_.

**Fig. 3:**
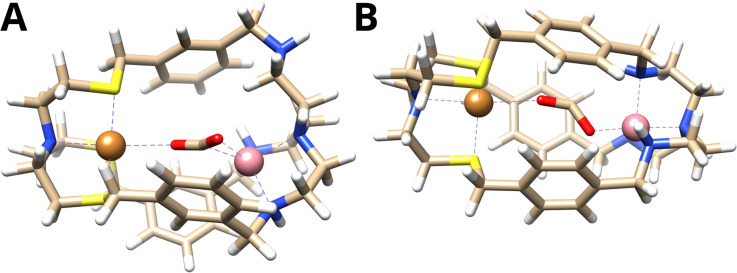
Binding mode of the CO_2_ radical anion within (A) Cu^I^Co^II^-{N^S^N^N^}_m_ and (B) Cu^I^Co^II^-{N^S^N^N^}_p_.

The computed binding mode of the CO_2_ radical anion for the *meta* and *para* analogue of the Cu^I^Co^II^ complex (Fig. 3 and S21, ESI†) also supports the significantly lowered activity of Cu^I^Co^II^-{N^S^N^N^}_p_. While the distance between the binding sites in {N^S^N^N^}_m_ is inherently smaller,^35^ it exhibits a higher flexibility and the corresponding metal complex can consequently adapt an even smaller bite length in the presence of various substrates (Table S3, ESI†). For example, owing to the flexible nature of Cu^I^Co^II^-{N^S^N^N^}_m_, the Cu–Co distance reduces from 6.20 to 4.60 Å for the accommodation of the CO_2_ radical anion, affording a dual μ_2_,η^2^_C,O_ binding mode. In contrast, the lowered flexibility of Cu^I^Co^II^-{N^S^N^N^}_p_ only allows for a deviation of the metal distance by 0.19 Å, binding the CO_2_˙^−^*via* its two oxo sites in a μ_2_,η^2^_O,O_ fashion, consequently preventing synergistic catalysis.

### Comparison with the octa-amino, homodinuclear cryptate Co^II^Co^II^-{N^N^N^N^}

To classify the activity of Cu^I^Co^II^-{N^S^N^N^}_m_ and compare it with Co^II^Co^II^-{N^N^N^N^}_m_ described by Lu *et al.*,^34^ we prepared the full-nitrogen system according to the described procedure and tested it as photocatalyst for the CO_2_ reduction under the herein stated conditions with 2 μM catalyst concentration. Small deviations within the photosystem set-up compared to the one used in literature might lead to a great difference in product outcome and make a direct comparison of the results difficult. In the herein employed photosystem, Co^II^Co^II^-{N^N^N^N^}_m_ achieved a production rate of 2.01 min^−1^ (11.6 μmol, TON of 2900, 24 h) and 6.51 × 10^−2^ min^−1^ (0.375 μmol, TON of 94, 24 h) for CO and H_2_, respectively, corresponding to a 97% selectivity for CO_2_-to-CO conversion (Fig. 2C). Notably, the activity of Cu^I^Co^II^-{N^S^N^N^}_m_ towards CO_2_-to-CO conversion is with 1.60 min^−1^ smaller with a maintained selectivity over H_2_ production as with Co^II^Co^II^-{N^N^N^N^}_m_. Although Cu^I^Co^II^-{N^S^N^N^}_m_ allows for the reduction of Co^II^ to Co^I^ at more anodic potential (−1.01 V *vs.* Fc^+/0^) compared to Co^II^Co^II^-{N^N^N^N^}_m_ (−1.15 V *vs.* Fc^+0^, one electron reduction to Co^I^Co^II33^), the LSV of the latter shows a drastic increase of current upon CO_2_ saturation after the Co^II^ reduction wave (Fig. S11B, ESI†). For Cu^I^Co^II^-{N^S^N^N^}_m_, a similar current is only observed at roughly 0.4 V more cathodic potentials, providing one reason for the lower activity compared to Co^II^Co^II^-{N^N^N^N^}_m_. Although a comparison of the proposed reaction mechanisms for the CO_2_-to-CO conversion with Cu^I^Co^II^-{N^S^N^N^}_m_ and the previously described Co^II^Co^II^-{N^N^N^N^}_m_ reveal that they follow different pathways for the actual reduction of CO_2_, the critical C–OH bond cleaving step is similar for both.^33^ Here, the assisting binding site, Cu^I^-{N^S^} or Co^II^-{N^N^} in case of Cu^I^Co^II^-{N^S^N^N^}_m_ and Co^II^Co^II^-{N^N^N^N^}_m_, respectively, plays an important role. Since Cu^I^-{N^S^} exhibits a higher electron density compared to Co^II^-{N^N^}, the binding affinity to the negatively charged OH^−^ is less pronounced. Thus, bond cleavage is promoted to a lower extent, consequently resulting in the lower activity of Cu^I^Co^II^-{N^S^N^N^}_m_. Furthermore, next to the synergistic pathway in Co^II^Co^II^-{N^N^N^N^}_m_, single-sited catalysis at both Co centres individually is conceivable as well, considerably enhancing the chance for CO_2_ reduction to occur. Due to the inactivity of the single Cu^I^-{N^S^} site, this is not the case for Cu^I^Co^II^-{N^S^N^N^}_m_.

At this point, we became curious on investigating the effect of incorporating two Co^II^ into {N^S^N^N^}_m_ on the photocatalytic CO_2_ reduction. Experiments with Co^II^Co^II^-{N^S^N^N^}_m_ however, revealed lower catalytic rates for CO_2_-to-CO conversion with 1.11 min^−1^ (6.39 μmol, TON of 1598, 24 h) compared to both, Co^II^Co^II^-{N^N^N^N^}_m_ and Cu^I^Co^II^-{N^S^N^N^}_m_. Same was for Zn^II^Co^II^-{N^S^N^N^}_m_ with 1.08 min^−1^ (6.21 μmol, TON of 1553, 24 h), despite the significantly improved catalysis observed for the corresponding Zn^II^Co^II^ complex of the full-nitrogen system {N_N_N_N_}_m_.^34^ Interestingly, the second metal seemingly does not promote the catalysis at all, since the observed rates are in the similar range as for monometallic Co^II^-{N^S^N^N^}_m_ (0.99 min ^−1^). The decreased catalytic rates and formation of a precipitate during irradiation together with a few reports on +II metal complexes revealing decreased activity for CO_2_-to-CO conversion or tendency to demetallation upon directs N/S exchange in the ligand under reductive conditions,^41–43^ let us to the conclusion that the lowered activity is due to decomposition of the {N^S^} site and consequently emphasises the importance of matching donor–acceptor properties of ligand and metal.

## Experimental

### General techniques

{N^S^N^N^}_m_ and {N^S^N^N^}_p_ as well as the corresponding metal complexes Cu^I^-{N^S^N^N^}_m_, Co^II^-{N^S^N^N^}_m_, Cu^I^Co^II^-{N^S^N^N^}_m_ and Cu^I^Co^II^-{N^S^N^N^}_p_ were synthesized according to a procedure developed previously within our group.^35^ For additional characterisation of the beforehand mentioned complexes, CHN experiments were measured with an Elementar vario MICRO cube. Co^II^Co^II^-{N^N^N^N^}_m_ was synthesised according to a literature-known procedure.^33^ Mass spectra were measured on a Advion expression^L^ instrument. IR measurements were performed on a Shimadzu IRTracer-100 attached with a Pike Miracle ATR unit and are reported in [cm^−1^]. UV/Vis/NIR spectra were recorded with a Shimadzu UV 1900i at 25 °C and are reported in [nm]. X-Band EPR spectra were recorded on a Bruker EMXplus X-band EPR spectrometer. Samples were frozen using a liquid helium recirculating cooling system provided by ColdEdge and were measured at 13 K. The solution in the tube was frozen in liquid nitrogen and kept frozen until measured. ^1^H NMR spectra for application of Evans' method were obtained on a Bruker DRX 400 MHz NMR spectrometer at room temperature in deuterated acetonitrile (MeCN-d_3_), followed by evaluation according to a literature-known procedure. The calculated spin-only effective moment was using the following equation1
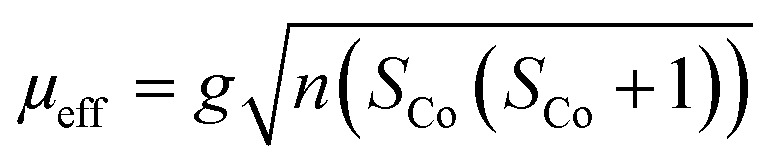
with *n* = number of Co^II^ centres, gyromagnetic ratio *g* = 2.00003 μ_B_ and spin at Co *S*_Co_ = 3/2 for hs Co^II^.^44–46^

### CHN analysis

#### Cu^I^Co^II^-{N^S^N^N^}_m_

Anal. calcd for [C_36_H_51_BCl_2_CoCuF_4_N_5_O_8_S_3_] + 2 MeOH: C 40.67, H 5.30, N 6.24; found: C 41.32, H 5.31, N 6.34.

#### Cu^I^-{N^S^N^N^}_m_

Anal. calcd for [C_36_H_51_BCuF_4_N_5_S_3_] 3H_2_O + 3 DCM: C 42.23, H 5.73, N 6.31; found: C 42.15, H 5.58, N 6.64.

#### Co^II^-{N^S^N^N^}_m_

Anal. calcd for [C_36_H_51_Cl_2_CoN_5_O_8_S_3_] + DCM + 2.5H_2_O: C 42.82, H 5.63, N 6.75; found: C 42.83, H 5.37, N 6.62.

#### Cu^I^Co^II^-{N^S^N^N^}_p_

Anal. calcd for [C_36_H_51_BCl_2_CoCuF_4_N_5_O_8_S_3_] + 2.5 MeOH + 0.5H_2_O: C 40.31, H 5.45, N 6.10; found: C 40.95, H 5.34, N 5.93.

### Synthesis of Co^II^Co^II^-{N^S^N^N^}_m_

{N^S^N^N^}_m_ (34.9 mg, 53.7 μmol) was dissolved in minimum amount of DCM. MeCN/MeOH (4 : 1) was added (2.5 mL), followed by the addition of Co(ClO_4_)_2_·6H_2_O (39.3 mg, 107 μmol) dissolved in 0.5 mL MeCN/MeOH (4 : 1) whereupon a direct colour change to brown was observed. The solution was stirred at room temperature for 24 h. Subsequently, the solvent was removed, the residue taken up in MeCN and filtered through a short silica column. After removal of the solvent, [Co^II^Co^II^{N^S^N^N^}_m_](ClO_4_)_4_ (Co^II^Co^II^-{N^S^N^N^}_m_) was obtained as a brown solid in 85% (53.2 mg, 45.6 μmol). ESI-MS: calcd for [Co^II^Co^II^-{N^S^N^N^}_m_(-3H)(MeCN)(OH)(Na)]^+^ m/z = 845.2; found: 844.9. Anal. calcd for [C_36_H_51_Cl_4_Co_2_N_5_O_16_S_3_] + 0.8 MeCN + 8H_2_O + 2 DCM: C 31.45, H 4.89, N 5.37; found: C 31.67, H 4.70, N 5.43. IR (ATR): *ν̃* = 3458, 3254, 2943, 2318, 2291, 1638, 1450, 1367, 1101, 930, 802 cm^−1^. UV/vis/NIR (MeCN): *λ*_max_ (ε in L mol^−1^ cm^−1^) = 384 (202 ± 2), 480 (114 ± 3), 587 (54 ± 1) nm. EPR (MeCN, 13 K): *g* = 4.29 (hs Co^II^).^47^ Evans method (MeCN-d_3_, 298 K): calcd for two hs Co^II^ μ_eff_ = 5.48 μ_B_; exp.: 5.64 μ_B_.

### Synthesis of Zn^II^Co^II^-{N^S^N^N^}_m_

{N^S^N^N^}_m_ (35.0 mg, 53.8 μmol) was dissolved in minimum amount of DCM. MeCN/MeOH (4 : 1) was added (2.5 mL), followed by the addition of Co(ClO_4_)_2_·6H_2_O (19.7 mg, 53.8 μmol) dissolved in 0.5 mL MeCN/MeOH (4 : 1) whereupon a direct colour change to light blue was observed. The solution was stirred at room temperature for 24 h. Subsequently, Zn(ClO_4_)_2_·6H_2_O (20.1 mg, 53.8 μmol) dissolved in 0.5 mL MeCN/MeOH (4 : 1) was added. Upon addition, the colour changed to beige and it was stirred at room temperature for 24 h. The solvent was removed, the residue taken up in MeCN and filtered through a short silica column. After removal of the solvent, [Zn^II^Co^II^{N^S^N^N^}_m_](ClO_4_)_4_ (Zn^II^Co^II^-{N^S^N^N^}_m_) was obtained as a beige solid in 76% (47.9 mg, 40.9 μmol). ESI-MS: calcd for [Zn^II^Co^II^-{N^S^N^N^}_m_(ClO_4_) (-2H)(MeCN)(Na)]^+^ m/z = 933.1; found: 933.0; [Zn^II^Co^II^-{N^S^N^N^}_m_(ClO_4_)(-H)(MeCN)(Na)(Li)]^+^ m/z = 941.1; found: 940.5; [Zn^II^Co^II^-{N^S^N^N^}_m_(2ClO_4_)(-2H)(Li)]^+^ m/z = 975.1; found: 975.9; calcd for [Zn^II^Co^II^-{N^S^N^N^}_m_(4ClO_4_)(H_2_O)]^+^ m/z = 1089.5; found: 1088.8. Anal. calcd for [C_36_H_51_Cl_4_CoN_5_O_16_S_3_Zn] + 1.5 MeCN + 6H_2_O + 2 DCM: C 32.58, H 4.77, N 6.02; found: C 32.25, H 4.54, N 6.11. IR (ATR): *ν̃* = 3460, 3246, 2941, 2317, 2290, 1637, 1450, 1365, 1098, 930, 802 cm^−1^. UV/vis/NIR (MeCN): *λ*_max_ (ε in L mol^−1^ cm^−1^) = 380 (163 ± 6), 480 (57 ± 7), 571 (25 ± 6) nm. EPR (MeCN, 13 K): *g* = 4.37 (hs Co^II^).^47^ Evans method (MeCN-d_3_, 298 K): calcd for one hs Co^II^ μ_eff_ = 3.87 μ_B_; exp.: 3.17 μ_B_.

### Photochemistry

The photocatalytic reduction of CO_2_ was performed at 25 °C in a 12 mL reactor sealed with a rubber septum containing 2 mL of the catalyst (0.5–2 μM), 0.4 mM [Ru(phen)_3_](PF_6_)_2_ as photosensitizer and 0.3 M TEOA as sacrificial electron donor in degassed MeCN or MeCN/H_2_O (9 : 1, 4 : 1 or 1 : 1 mixtures). The solution was saturated with either Ar, ^12^CO_2_ or ^13^CO_2_ and was illuminated with blue LED light (*λ* = 450 nm, 1200 mcd, 0.8 cm^2^ irradiation area) for minimum 24 h and maximum 56 h. For measurements in the absence of light, the reaction was performed in a black chamber. To identify the composition of the gas phase, 50 μL of the headspace were analysed two-hourly by GC-BID *via* hand injection. To analyse the generated liquid products after photocatalysis, 1 mL of the solution was acidified with 100 μL conc. H_2_SO_4_ and analysed *via* headspace GC-MS (Fig. S3, ESI†). To verify the absence of formate/formic acid, the liquid phase was further analysed for propyl formate after derivatisation with *n*-propanol. Therefore, 400 μL sample were treated with 500 μL *n*-propanol and 100 μL *p*-toluene sulfonic acid and analysed *via* headspace GC-MS (Fig. S4, ESI†). Each photocatalytic reaction was repeated at least three times to confirm the reliability of the data. A summary of the determined data can be found in the ESI (Table S4†).

### Determination of quantum yield

The quantum yields of the photocatalytic CO_2_ reduction experiments were determined using the equation2
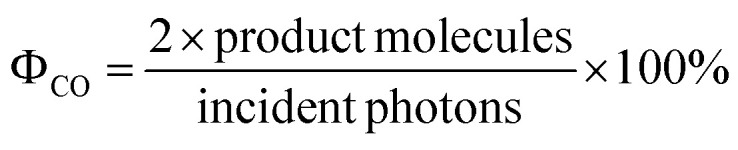
where the product molecules were quantified using a calibrated GC-MS or GC-BID system and the number of incident photons was determined using potassium ferrioxalate (K_3_[Fe^III^(C_2_O_4_)_3_]) as chemical actinometer at 25 °C.^48^ Accordingly, the photon flux was determined to be 8.92 × 10^16^ s^−1^.

### Quantification of CO_2_RR products

Quantification of the headspace gas composition of the photochemical cell was performed using a Shimadzu GC-2010 Pro equipped with a Shimadzu BID-2010 Plus barrier discharge ionization detector (BID). Gas phase separation was performed *via* hand injection using a SPL injector and a Carboxen 1010 PLOT fused silica capillary GC column (L × I.D. 30 m × 0.32 mm, average thickness 15 μm). Solvents were cut off previously with a SH-Rxi-1ms fused silica capillary GC column (L × I.D. 30 m × 0.32 mm, average thickness 1.0 μm) and were deflected to a second detector (TCD). Helium was used as carrier gas. The following gaseous products/components were assayed *via* the GC-BID system: H_2_, O_2_, N_2_, CO, CH_4_, C_2_H_4_, C_2_H_6_. Quantification of the liquid phase composition of the photochemical cell was performed using a Shimadzu GCMS-QP2020 system equipped with a MS detector. Phase separation was performed *via* headspace analysis using a SH-Rtx-200 ms fused silica capillary GC column (L × I.D. 30 m × 0.25 mm, average thickness 1 μm). Helium was used as carrier gas. The following liquid products/components were assayed *via* the GCMS system: methanol, ethanol, propanol, formate/formic acid, acetate/acetic acid, propionate/propionic acid, acetaldehyde and propionaldehyde. The given values are averaged over three experiments with typical uncertainties of ±2–8%.

### High-performance liquid chromatography

Analytical HPLC was performed using a Knauer Smartline setup with a four-wavelength detector and a dynamic mixing chamber with reversed-phase chromatography column (Nucleodur C4 gravity, 5 μm, 125 × 4 mm, flow rate 1 mL min^−1^). MiliQ-water with 0.1% trifluoroacetic acid (buffer A) and MeCN with 0.1% trifluoroacetic acid (buffer B) were used as eluents. For the analysis, a linear gradient from 99% to 1% buffer A was driven over 15 min.

### Particle size measurement

Particle sizes were measured using a Shimadzu SALD-2300 laser diffraction particle size analyzer equipped with a SALD-BC23 Batch cell. Therefore, 1 mL of the irradiated photocatalytic solution was added to the measurement cell containing 12 mL MeCN/H_2_O (4 : 1). The obtained particle sizes were calculated using the Fraunhofer approximation.

### Electrochemistry

The electrochemical studies were performed using a PalmSens3 or a PalmSens4 potentiostat in a standard three-electrode setup. A glassy carbon electrode was used as working electrode (WE), an Ag wire as pseudo-reference electrode (PRE) and a Pt wire as counter electrode (CE). The working electrode was prepared by successive polishing with 1.0 and 0.3 μm sandpaper and subsequent sonication in MeCN for 10 min. Tetrabutylammonium hexafluorophosphate ([^*n*^Bu_4_N]PF_6_, 0.1 M) was used as electrolyte in all electrochemical measurements either in anhydrous MeCN or a mixture of MeCN/H_2_O (4 : 1). Prior to each experiment, the electrochemical cell was degassed with Ar for 10 min and an Ar or CO_2_ atmosphere was maintained throughout the measurement. All cyclic voltammograms were recorded at a scan rate of 100 mV s^−1^ and after every experiment all pseudo-referenced potentials were referenced against the ferrocenium/ferrocene couple (Fc^+/0^).

### Luminescence quenching

Luminescence lifetimes were measured on an Edinburgh Instruments Lifespec II spectrometer with a 472 nm pulsed diode laser (pulse width 75.5 ps).

### Computational studies

All reported calculations were carried out with ORCA program package in its version 5.0.3.^49^ The meta-hybrid TPSSh functional^50^ in conjunction with the ZORA-def2-TZVP(-f) basis set^51^ was used during geometry optimisations and final energy evaluations. Grimme's semiempirical van der Waals corrections with the Becke-Johnson damping (D3BJ) were employed to describe dispersive and non-covalent interactions. Scalar relativistic effects were approximated through the zeroth order regular approximation (ZORA).^52–54^ The resolution-of-identity (RI) along with chain-of-spheres algorithms (COSX) were used during the SCF cycles to accelerate the computation of two-electron integrals, while utilising the SARC/J basis set for the former approximation as an auxiliary basis.^55–60^ Solvent effects were incorporated implicitly within the Conductor-like Polarizable Continuum Model (C-PCM).^61,62^ All structures were fully optimised without symmetry constraints. Normal mode analysis was performed for all reported structures to identify the nature of the ground state and transition state structures. Free energies were reported in kcal mol^−1^ at 1 atm and 298 K. An electrode potential was applied by adding −*eΦ* with a value of *Φ* = 1.12 V to the electronic energy for each added electron. The Gibbs free energy of a proton in acetonitrile was taken to be −260.2 kcal mol^−1^.

## Conclusions

In summary, we herein describe Cu^I^Co^II^-{N^S^N^N^}_m_ as efficient catalyst for the visible light-driven reduction of CO_2_ achieving a CO production rate of 1.60 min^−1^ with a high selectivity of 98% over H_2_ production. While Cu^I^Co^II^-{N^S^N^N^}_m_ is a stable, homogeneous catalyst, the photosystem durability is limited to 24 h due to photosensitiser degradation, but can be reactivated through addition of new [Ru(phen)_3_](PF_6_)_2_. Experiments with the mononuclear analogues of Cu^I^Co^II^-{N^S^N^N^}_m_ clearly reveal that the simultaneous presence of both metals has a beneficial influence on the amount of CO produced. While with Cu^I^-{N^S^N^N^}_m_ nearly no CO_2_ reduction was observed, Co^II^-{N^S^N^N^}_m_ produced CO but exhibits a smaller activity than the dinuclear cryptate (0.99 min^−1^). Computational studies revealed that in case of Co^II^-{N^S^N^N^}_m_ single Co^II^-sited catalysis is responsible for CO_2_ reduction and that the higher activity with Cu^I^Co^II^-{N^S^N^N^}_m_ is indeed based on synergistic effects between Cu and Co. Furthermore, experimental and computational studies with Cu^I^Co^II^-{N^S^N^N^}_p_ as catalyst show that the increased bite length together with the decreased flexibility of the *para*-substituted cryptand prevent synergistic CO_2_ reduction, thus leading to a significantly smaller catalytic rate. Moreover, compared to Co^II^Co^II^-{N^N^N^N^}_m_, the exchange of one Co^II^-{N^N^} site by Cu^I^-{N^S^} in Cu^I^Co^II^-{N^S^N^N^}_m_ is accompanied by a slight decrease in activity due to the smaller binding affinity of the Cu^I^-{N^S^} site to OH^−^ which is especially important in the critical C–OH cleaving step. The herein presented results thus provide important insight into the design of bimetallic catalysts and highlight the importance of the interplay of the metal distance as well as binding strength to CO_2_ for an efficient C–O bond cleavage and synergistic catalysis.

## Data availability

Supplemental figures, tables and relevant data including Cartesian coordinates of all computationally investigated structures have been provided in the ESI.†

## Author contributions

J. J. planned and performed synthesis and characterisation of the catalysts, photocatalytic and electrochemical experiments, degradation studies and prepared the original manuscript. E. B. B. carried out all theoretical computations and was involved in writing the manuscript. J. W. performed the luminescence quenching experiments. O. S. W. supervised the luminescence quenching experiments and revised the manuscript. Ma. R. aided in interpreting the results and planning of photocatalytic experiments and revised the manuscript. Mi. R. conceived and supervised all theoretical calculations and was involved in the writing of the manuscript. U.-P. A. conceived the whole research, supervised photocatalytic experiments and finalised the manuscript.

## Conflicts of interest

There are no conflicts to declare.

## Supplementary Material

SC-014-D3SC02679E-s001
