# Supplementary material for: A CuICoII cryptate for the visible light-driven reduction of CO2
Source: Chem Sci. 2023 Oct 27;14(44):12774–83. doi: 10.1039/d3sc02679e (PMC10646873; doi:10.1039/d3sc02679e)
Supplement: SC-014-D3SC02679E-s001 [file SC-014-D3SC02679E-s001.pdf]

Electronic Supplementary Material (ESI) for Chemical Science.
This journal is © The Royal Society of Chemistry 2023

Supporting Information

A $Cu^{I}Co^{II}$ cryptate for the visible-light driven reduction of CO_2

Julia Jökel,[a] Esma Birsen Boydas,[b] Joël Wellauer,[c] Oliver S. Wenger,[c] Marc Robert,[d,e] Michael Roemelt*[b] and Ulf-Peter Apfel*[a,f]

[a]Fraunhofer UMSICHT, Osterfelder Str. 3, 46047 Oberhausen, Germany. E-mail: ulf-peter.apfel@umsicht.fraunhofer.de, ulf.apfel@rub.de

[b]Institute of Chemistry, Humboldt-Universität zu Berlin, Brook-Taylor Str. 2, 12489 Berlin, Germany.

[c]Department of Chemistry, University of Basel, St. Johanns-Ring 19, 4056 Basel, Switzerland.

[d]Université Paris Cité, Laboratoire d’Electrochimie Moléculaire, CNRS, F-75013 Paris, France.

[e]Institut Universitaire de France (IUF), F-76006 Paris, France.

[f] Inorganic Chemistry I, Ruhr-Universität Bochum, Universitätsstr. 150, 44801 Bochum, Germany.

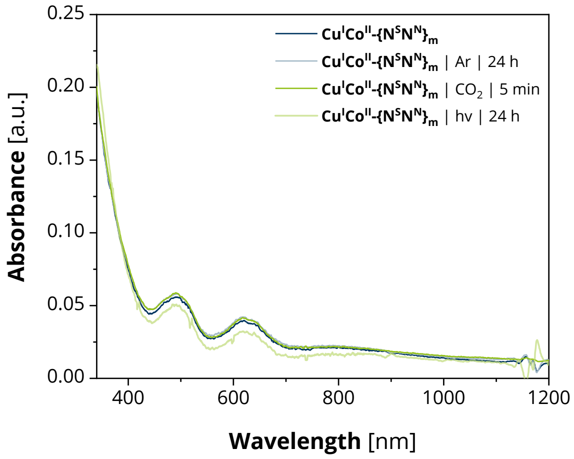

Figure S1 UV/vis/NIR spectra of **Cu^{I}Co^{II}-{N^{S}N^{N}}$_m$** in MeCN (0.6 mM) under Ar (dark blue), after 24 h under Ar in solution (light blue), purging with CO_2 for 5 min (green) and after 24 h irradiation under Ar (λ = 450 nm, 1200 mcd) (light green).

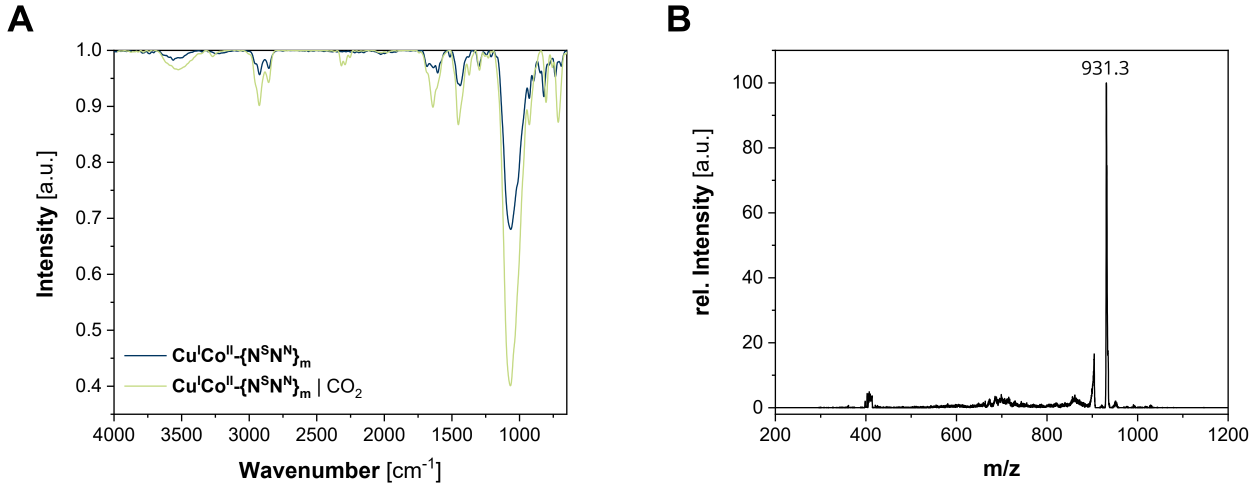

Figure S2 (A) IR spectra of **Cu^{I}Co^{II}-{N^{S}N^{N}}$_m$** in the absence and presence of CO_2. (B) ESI-MS spectrum of a CO_2-purged MeCN/H_2O (4:1) solution of **Cu^{I}Co^{II}-{N^{S}N^{N}}$_m$**.

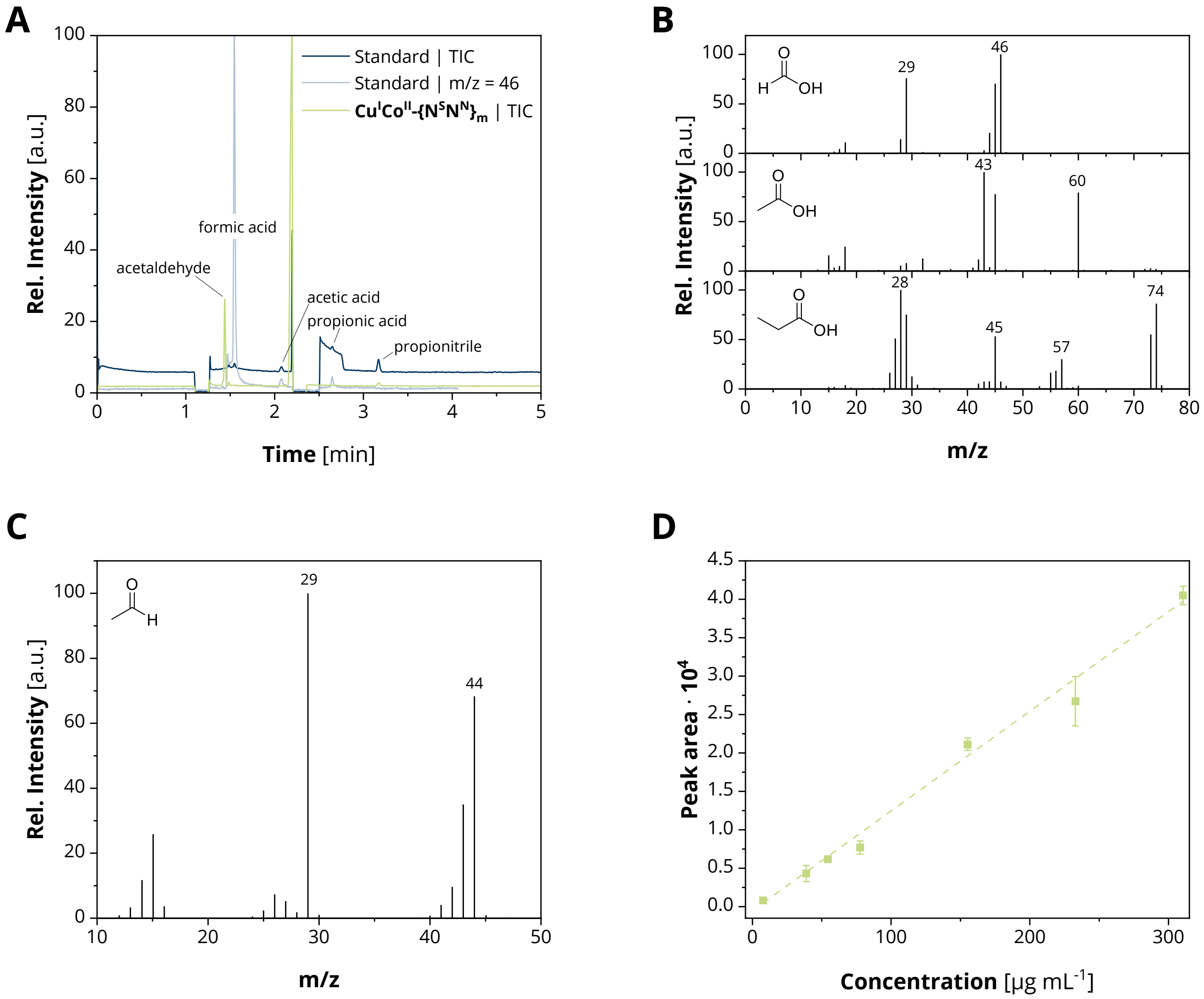

Figure S3 (A) GCMS traces of the calibration standard (total ion count and mass count at m/z = 46) containing 38.8 µg mL^{-1} formic acid, acetic acid and propionic acid in $MeCN/H_2O$ (4:1) as well as of the liquid phase of the photochemical cell containing 2 µM **$Cu^{I}Co^{II}$-{$N^{S}N^{N}$}$_{m}$**, 0.4 mM $[Ru(phen)_3](PF_6)_2$ and 0.3 M TEOA in $MeCN/H_2O$ (4:1) after 24 h irradiation with blue LED light (λ = 450 nm, 1200 mcd, irradiation area 0.8 cm^2). In each case, 1 mL sample was acidified with 100 µL conc. H_2SO_4 before measurement. (B) Mass spectra of formic acid, acetic acid and propionic acid from GCMS measurement of the calibration standard shown in (A). (C) Mass spectrum of acetaldehyde from GC-MS measurement of the photosystem shown in (A). (D) Calibration curve from GC-MS analysis of 1 mL calibration standards containing formic acid (7.76, 38.8, 54.3, 77.6, 155,233, 310 µg mL^{-1}) in $MeCN/H_2O$ (4:1) acidified with 100 µL conc. H_2SO_4.

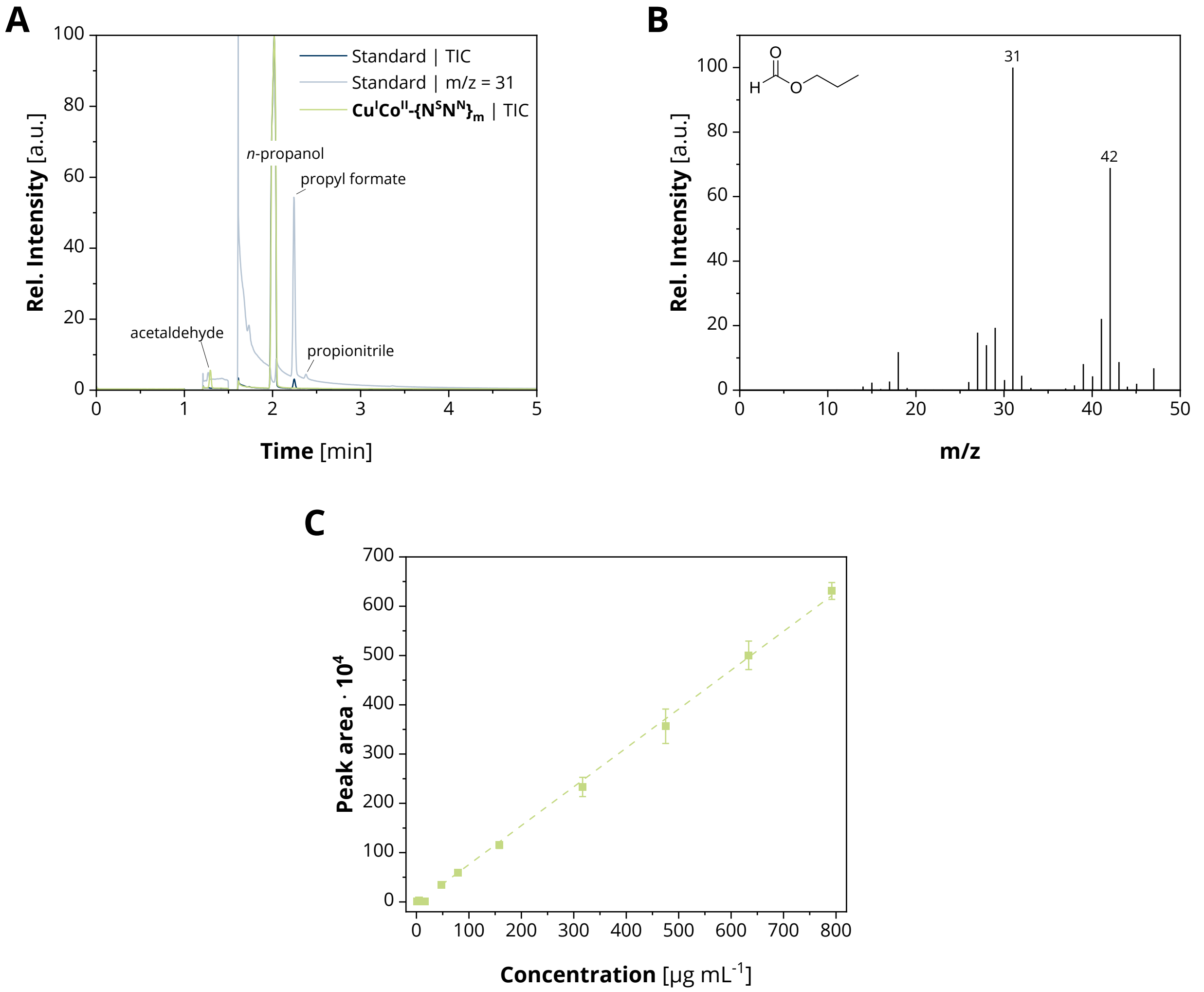

Figure S4 (A) GCMS traces of the calibration standard (total ion count and mass count at m/z = 41) containing 79.2 µg mL^{-1} formic acid derivatised with *n*-propanol to propyl formate in MeCN/H_2O (4:1) as well as of the liquid phase of the photochemical cell containing 2 µM **Cu^{I}Co^{II}-{N^{S}N^{N}}$_{m}$**, 0.4 mM [Ru(phen)$_3$](PF$_6$)$_2$ and 0.3 M TEOA in MeCN/H_2O (4:1) after 24 h irradiation with blue LED light (λ = 450 nm, 1200 mcd, irradiation area 0.8 cm^2). In each case, 400 µL sample was treated with 500 µL *n*-propanol and 100 µL 10% aq. *p*-toluene sulfonic acid. (B) Mass spectrum of propyl formate from GCMS measurement of the calibration standard shown in (A). (C) Calibration curve from GC-MS analysis of 400 µL calibration standard containing formic acid (1.58, 5.00, 15.8, 47.5, 79.2, 158, 317, 475, 634, 792 µg mL^{-1}), 500 µL *n*-propanol and 100 µL 10% aq. *p*-toluene sulfonic acid in MeCN/H_2O (4:1).

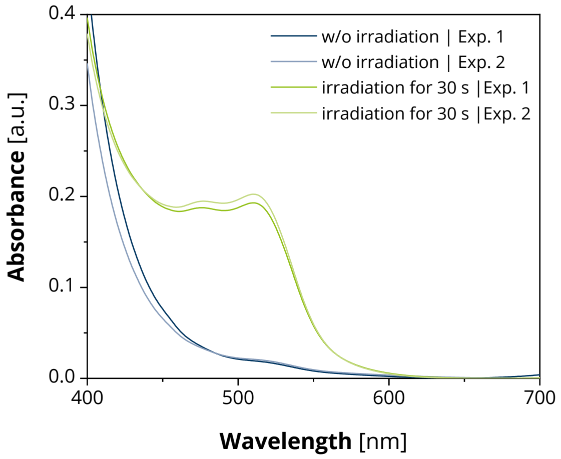

Figure S5 Determination of the number of incident photons using $K_3[Fe(C_2O_4)_3]\cdot 3H_2O$ as chemical actinometer.[1] Therefore, 2 mL containing 0.15 M $K_3[Fe(C_2O_4)_3]\cdot 3H_2O$ in 0.05 M H_2SO_4 were either irradiated with visible light (λ = 450 nm, 1200 mcd, irradiation area 0.8 cm^2) for 30 s or kept in the dark. Of both samples, 36 µL were subsequently added to 0.4 mL of 0.1% phenanthroline in 0.5 M H_2SO_4 buffered with sodium acetate (2.75 M) and diluted with 4.564 mL water. Each experiment was repeated two times. The absorbance was subsequently measured (blue: dark sample, green: irradiated sample) and the photon flux was determined using the literature-known procedure.[1]

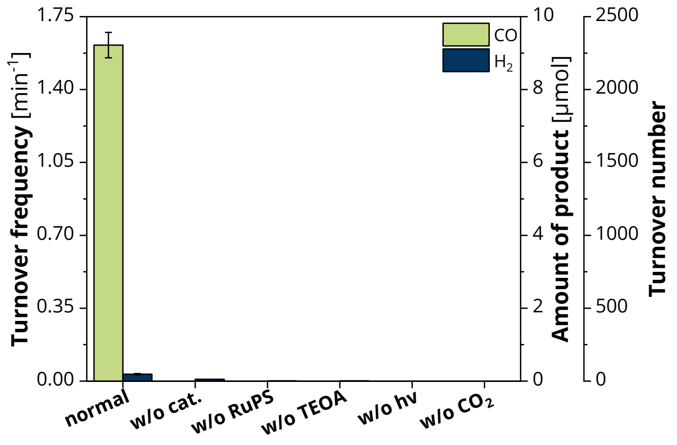

Figure S6 Photocatalytic evolution of CO (green) and H_2 (blue) after 24 h catalysed by **Cu^ICo^{II}-{N^SN^N}$_m$** (2 µM) in a CO_2-saturated MeCN/H_2O (4:1) solution containing 0.4 mM $[Ru(phen)_3](PF_6)_2$ and 0.3 M TEOA under irradiation with blue LED light (λ = 450 nm, 1200 mcd, irradiation area 0.8 cm^2) as well as a series of blind experiments in which one component described above was omitted individually.

[1] P. G. Alsabeh, A. Rosas-Hernández, E. Barsch, H. Junge, R. Ludwig and M. Beller, *Catal. Sci. Technol.*, 2016, **6**, 3623–3630.

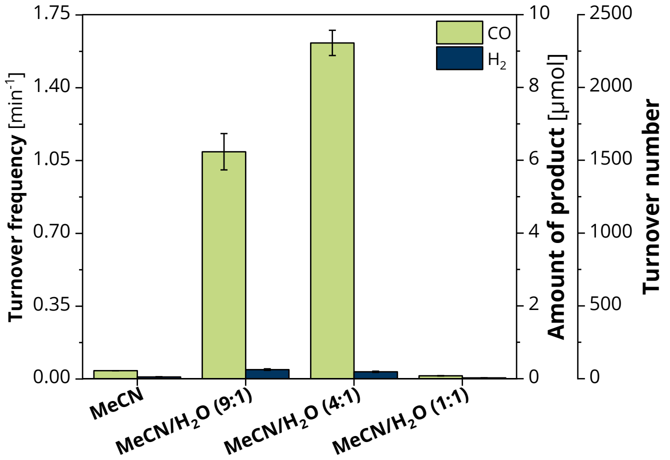

Figure S7 Photocatalytic evolution of CO (green) and H_2 (blue) after 24 h catalysed by **$Cu^{I}Co^{II}$-{$N^{S}N^{N}$}$_m$** (2 µM) in the presence of 0.4 mM $[Ru(phen)_3](PF_6)_2$ and 0.3 M TEOA under irradiation with blue LED light (λ = 450 nm, 1200 mcd, irradiation area 0.8 cm^2) in different mixtures of CO_2-saturated MeCN/H_2O (only MeCN, 9:1, 4:1, 1:1).

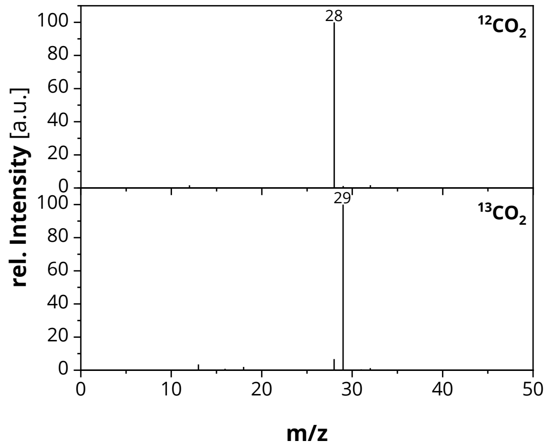

Figure S8 Mass spectrum of the generated gas from the photocatalytic experiment catalysed by **$Cu^{I}Co^{II}$-{$N^{S}N^{N}$}$_m$** (2 µM) in a $^{12}CO_2$- and $^{13}CO_2$-saturated MeCN/H_2O (4:1) solution containing 0.4 mM $[Ru(phen)_3](PF_6)_2$ and 0.3 M TEOA under irradiation with blue LED light (λ = 450 nm, 1200 mcd, irradiation area 0.8 cm^2) after 24 h.

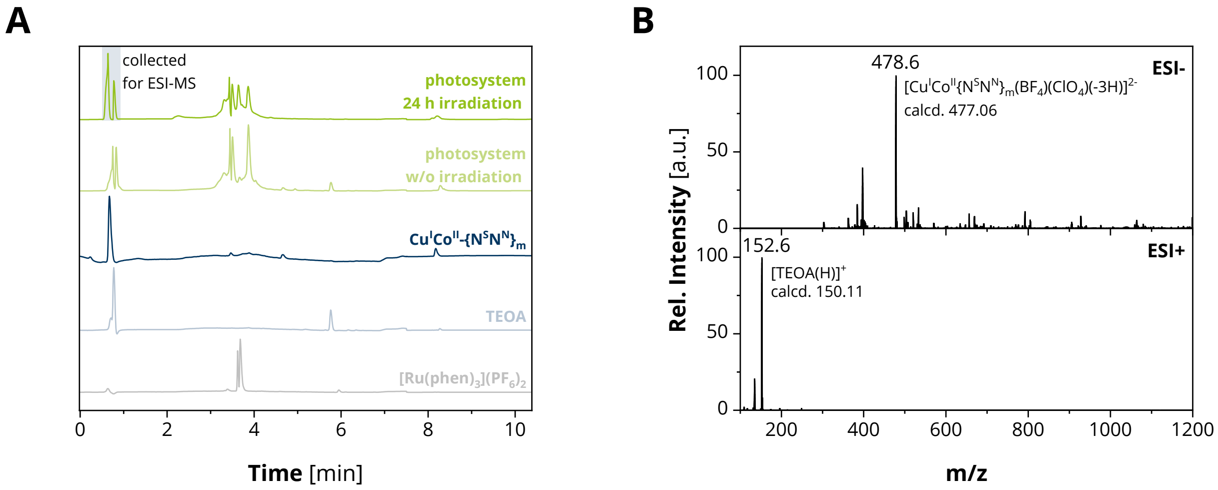

Figure S9 (A) HPLC traces of 1 mg/mL [Ru(phen)$_3$](PF$_6$)$_2$, TEOA and **Cu^{I}Co^{II}-{N^{S}N^N}$_m$** in MeCN/H_2O (4:1) as well as of the photocatalytic solutions containing 2 mM **Cu^{I}Co^{II}-{N^{S}N^N}$_m$**, 0.4 mM [Ru(phen)$_3$](PF$_6$)$_2$ and 0.3 M TEOA in MeCN/H_2O (4:1) without irradiation and after 24 h irradiation with blue LED light (λ = 450 nm, 1200 mcd, irradiation area 0.8 cm^2). (B) ESI-MS (top: negative ion mode, bottom: positive ion mode) of the catalyst and TEOA containing fraction collected during HPLC analysis shown in (A).

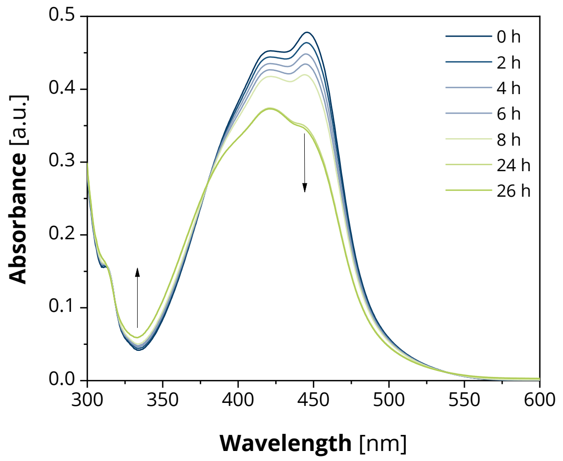

Figure S10 UV/vis spectrum of 0.02 mM [Ru(phen)$_3$](PF$_6$)$_2$ in MeCN/H_2O (4:1) during the time course of blue LED light irradiation (λ = 450 nm, 1200 mcd, irradiation area 0.8 cm^2).

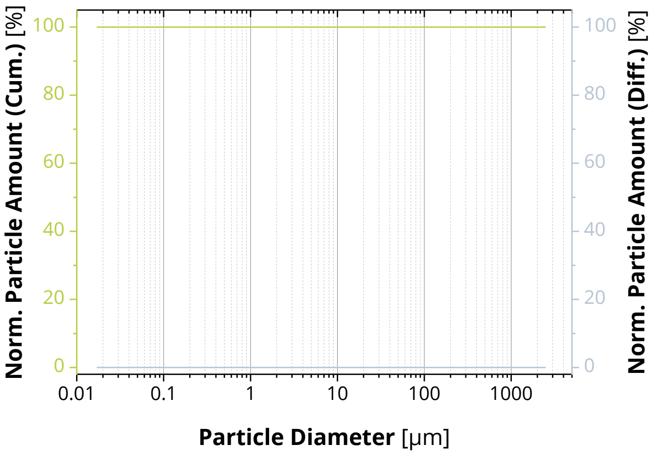

Figure S11 Particle size analysis of the photocatalytic solution containing 2 µM **Cu^I^Co^II^-{N^S^N^N^}~m~**, 0.4 mM $[Ru(phen)_3](PF_6)_2$ and 0.3 M TEOA in $MeCN/H_2O$ (4:1) after 24 h irradiation by blue LED light (λ = 450 nm, 1200 mcd, irradiation area 0.8 cm^2) by laser diffraction.

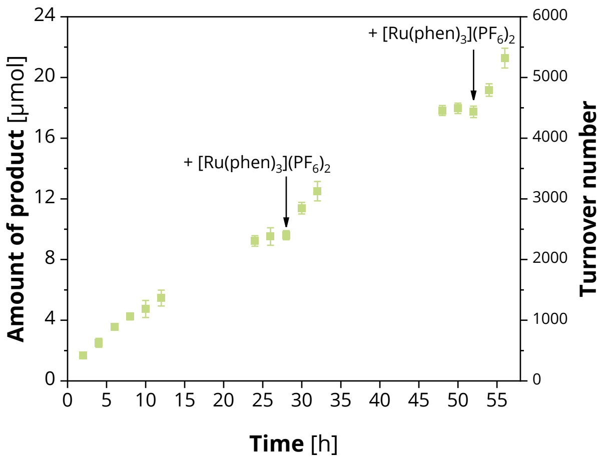

Figure S12 Amount of CO generated from the photocatalytic experiment catalysed by 2µM **Cu^ICo^{II}-{N^SN^N}$_m$** within a CO_2-saturated $MeCN/H_2O$ (4:1) solution containing 0.4 mM $[Ru(phen)_3](PF_6)_2$ and 0.3 M TEOA under irradiation with blue LED light (λ = 450 nm, 1200 mcd, irradiation area 0.8 cm^2) during 2-56 h. After 26 and 52 h, fresh $[Ru(phen)_3](PF_6)_2$ (50 µL of a 16 mM solution) was added to the completed catalytic system for reactivation.

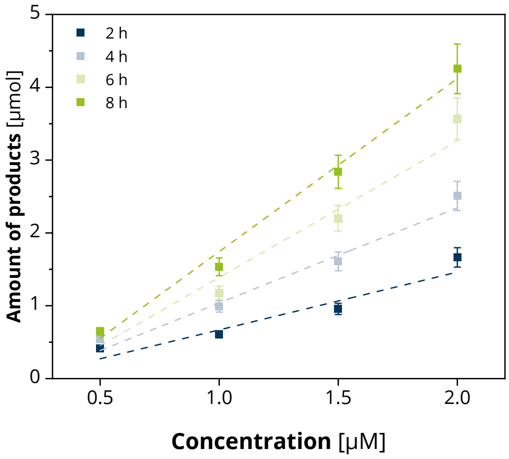

Figure S13 Amount of CO generated from the photocatalytic experiment catalysed within a CO_2-saturated MeCN/H_2O (4:1) solution containing 0.4 mM [Ru(phen)$_3$](PF$_6$)$_2$ and 0.3 M TEOA under irradiation with blue LED light (λ = 450 nm, 1200 mcd, irradiation area 0.8 cm^2) during 2-24 h in dependence of the concentration of the catalyst **Cu^{I}Co^{II}-{N^{S}N^N}$_m$** (0.5, 1.0, 1.5 and 2 µM).

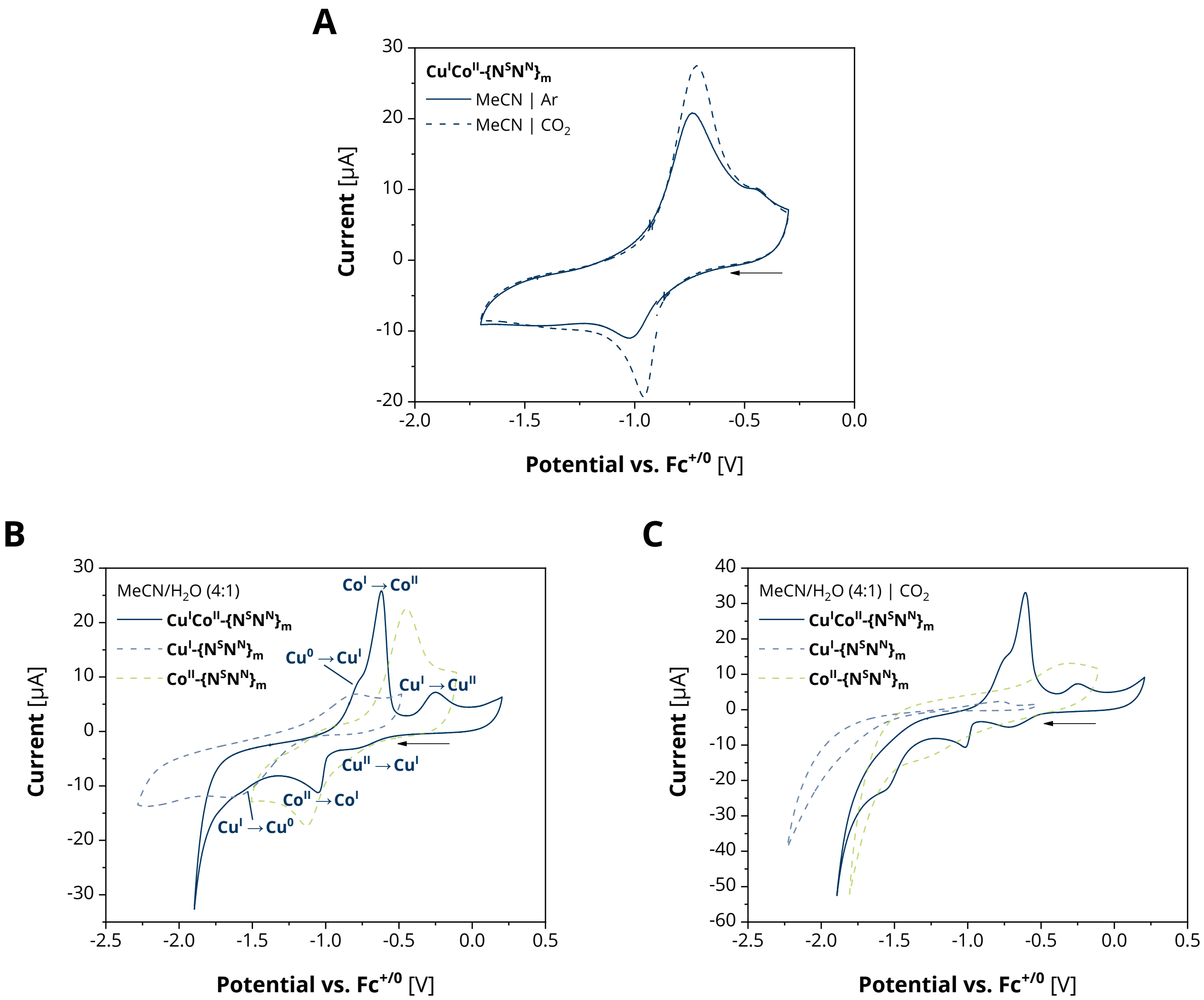

Figure S14 Cyclic voltammograms of (A) 1mM **Cu^{I}Co^{II}-{N^{S}N^N}$_m$** in MeCN under Ar or CO_2 atmosphere and of 1 mM **Cu^I-{N^{S}N^N}$_m$** (dashed light blue), **Co^{II}-{N^{S}N^N}$_m$** (dashed green) and **Cu^{I}Co^{II}-{N^{S}N^N}$_m$** (blue) in MeCN/H_2O (4:1) with 0.1 M [^{n}Bu$_4$N]PF$_6$ as supporting electrolyte with 100 mV s^{-1} under Ar atmosphere (B) or in the presence of CO_2 (C).

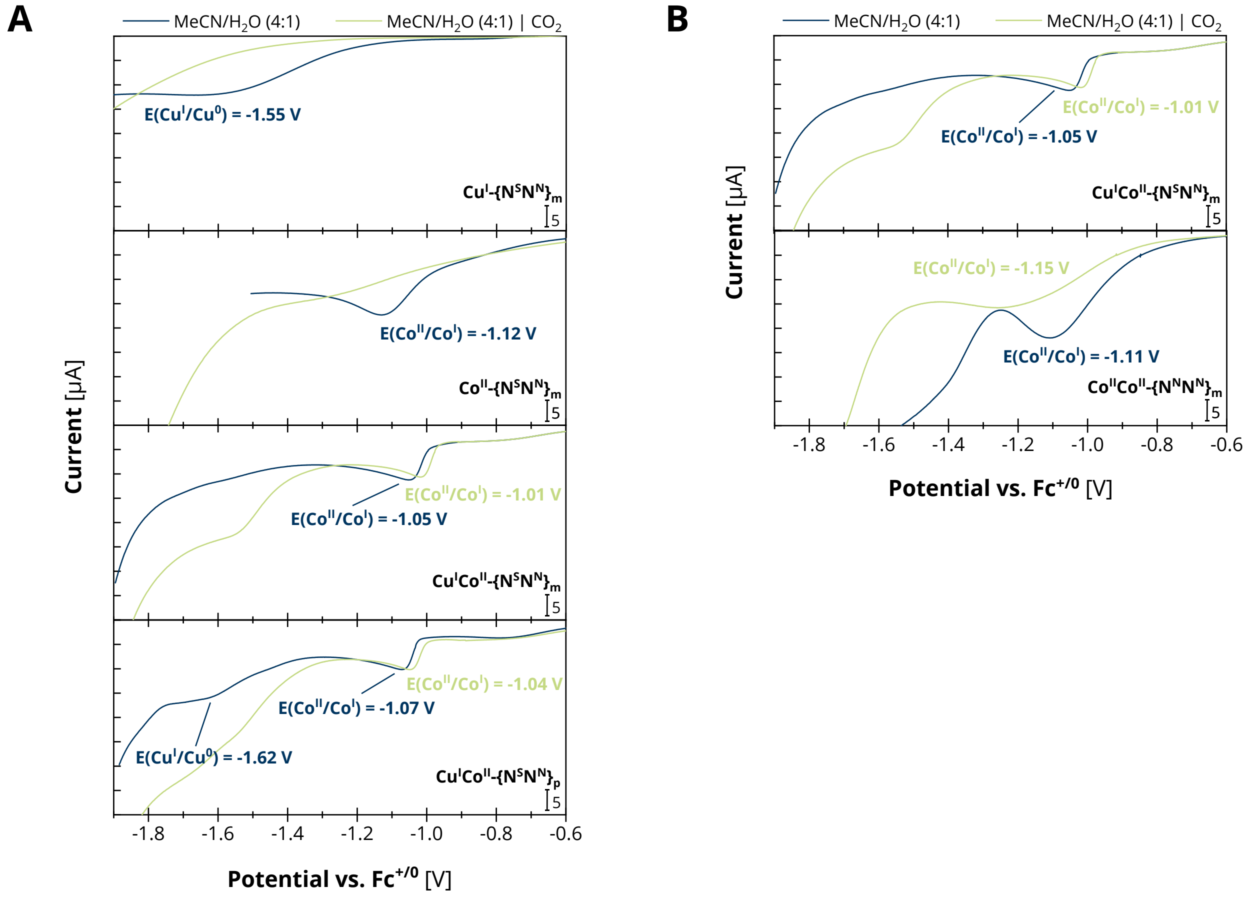

Figure S15 Linear sweep voltammograms of the herein investigated metal complexes (1 mM) in MeCN/H_2O (4:1) with 0.1 M [^{n}Bu_4N]PF_6 as supporting electrolyte with 100 mV s^{-1} under inert conditions (blue) or in the presence of CO_2 (green).

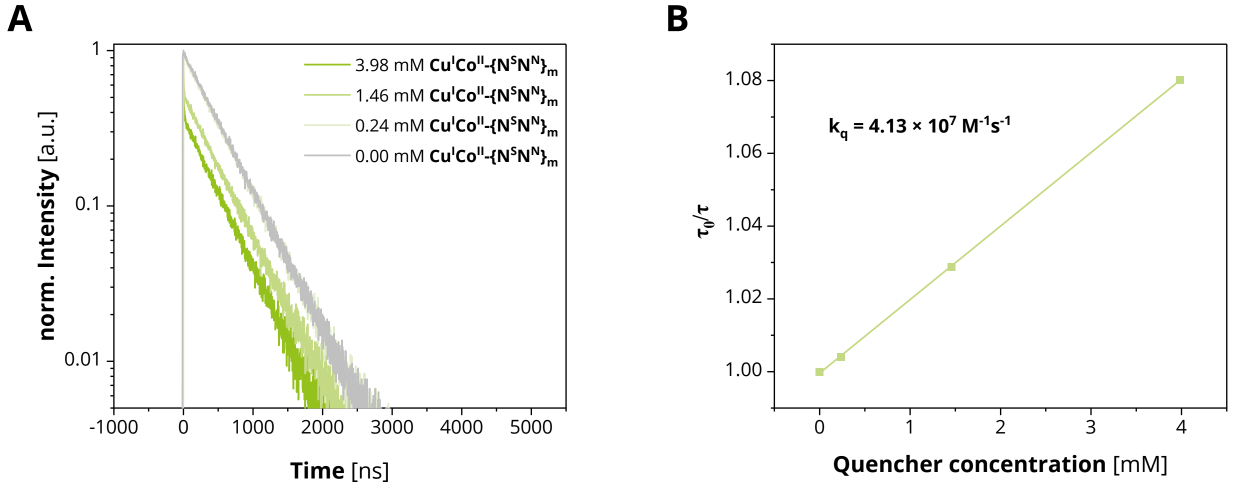

Figure S16 Luminescence quenching of at 472 nm excited [Ru(phen)$_3$](PF_6)$_2$ by **Cu^{I}Co^{II}-{N^{S}N^N}$_m$** in MeCN. (A) The emission decay was monitored at 620 nm in the absence (grey) and in the presence of different concentrations of **Cu^{I}Co^{II}-{N^{S}N^N}$_m$** (green). (B) Stern-Volmer plot with the calculated corresponding quenching rate constant k_q.

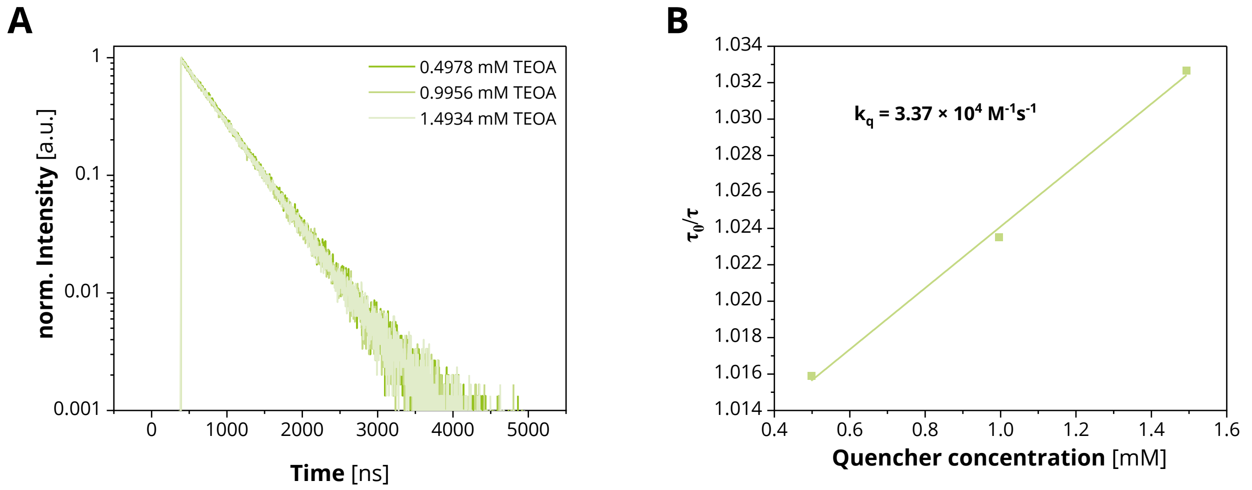

Figure S17 Luminescence quenching of at 472 nm excited $[Ru(phen)_3](PF_6)_2$ by TEOA in MeCN. (A) The emission decay was monitored at 620 nm in the presence of different concentrations of TEOA. (B) Stern-Volmer plot with the calculated corresponding quenching rate constant k_q.

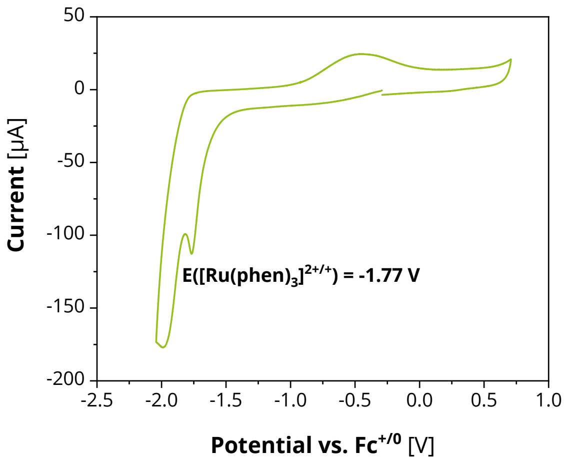

Figure S18 Cyclic voltammogram of $[Ru(phen)_3](PF_6)_2$ in MeCN/H_2O (4:1) with 0.1 M $[^nBu_4N]PF_6$ as supporting electrolyte with 100 mV s^{-1} under inert conditions.

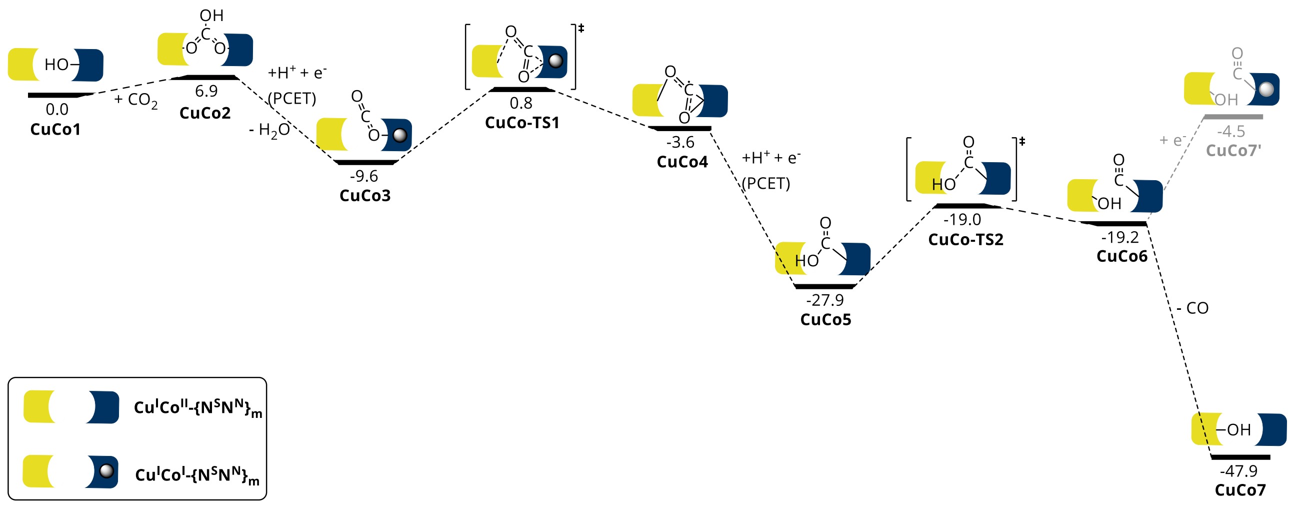

Figure S19 Proposed reaction mechanism for CO_2 reduction with **Cu^{I}Co^{II}-{N^{S}N^N}$_m$**. Energies are given in kcal mol^{-1}.

Table S1 Redox potential of the Co$^{II/I}$ couple within **Cu^{I}Co^{II}-{N^{S}N^N}$_m$** in the presence of various small molecules/anions or of the empty cavity.

	**Redox potential [V]**
empty	-0.98
CO	-0.19
CO_2	-0.77
MeCN	-1.27
OH^-	-1.90

Table S2 Binding energy of CO to the Co-site of **Cu^{I}Co^{II}-{N^{S}N^N}$_m$** in the initial or single-reduced state.

	**Binding energy [kcal mol^{-1}]**
Cu^{I}Co^{II}	-9.58
Cu^{I}Co^I	27.8

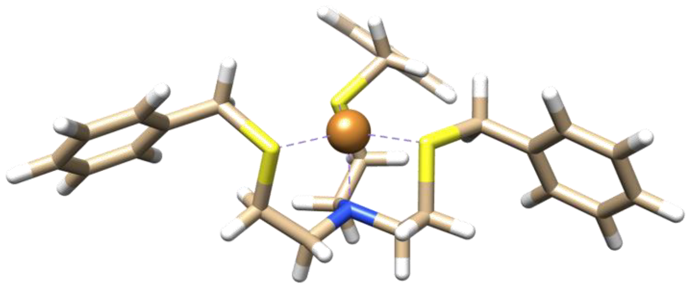

Figure S20 Mononuclear model system Cu^I-{N^S}.

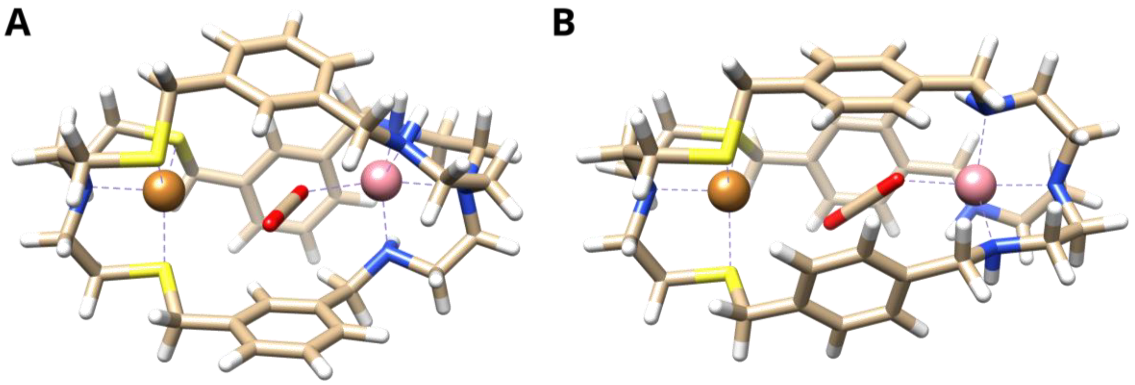

Figure S21 CO_2 binding modes within (A) **Cu^{I}Co^{II}-{N^{S}N^{N}}$_m$** and (B) **Cu^{I}Co^{II}-{N^{S}N^{N}}$_p$** in the initial, non-reduced state.

Table S3 Cu-Co distance in **Cu^{I}Co^{II}-{N^{S}N^{N}}$_m$** and **Cu^{I}Co^{II}-{N^{S}N^{N}}$_p$** in the empty cavity or in the presence of various small molecules or anions bound to one or both metals.

	**d [Å]**	
	**Cu^{I}Co^{II}-{N^{S}N^{N}}$_m$**	**Cu^{I}Co^{II}-{N^{S}N^{N}}$_p$**
empty	6.21	6.51
CO_2	5.87	6.62
$CO_2^{\cdot-}$	4.60	6.32
MeCN	5.94	6.66
OH^-	5.57	6.06
HCO_3^-	5.45	6.19

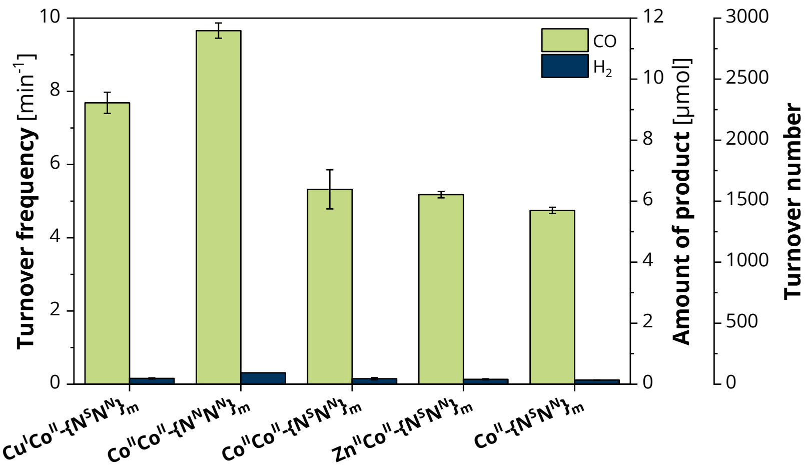

Figure S22 Photocatalytic evolution of CO (green) and H_2 (blue) after 24 h catalysed by **Cu^{I}Co^{II}-{N^{S}N^{N}}$_m$**, **Co^{II}Co^{II}-{N^{N}N^{N}}$_m$**, **Co^{II}Co^{II}-{N^{S}N^{N}}$_m$**, **Zn^{II}Co^{II}-{N^{S}N^{N}}$_m$** and **Co^{II}-{N^{S}N^{N}}$_m$** (2 µM) in the presence of 0.4 mM $[Ru(phen)_3](PF_6)_2$ and 0.3 M TEOA under irradiation with blue LED light (λ = 450 nm, 1200 mcd, irradiation area 0.8 cm^2) in CO_2-saturated $MeCN/H_2O$ (4:1).

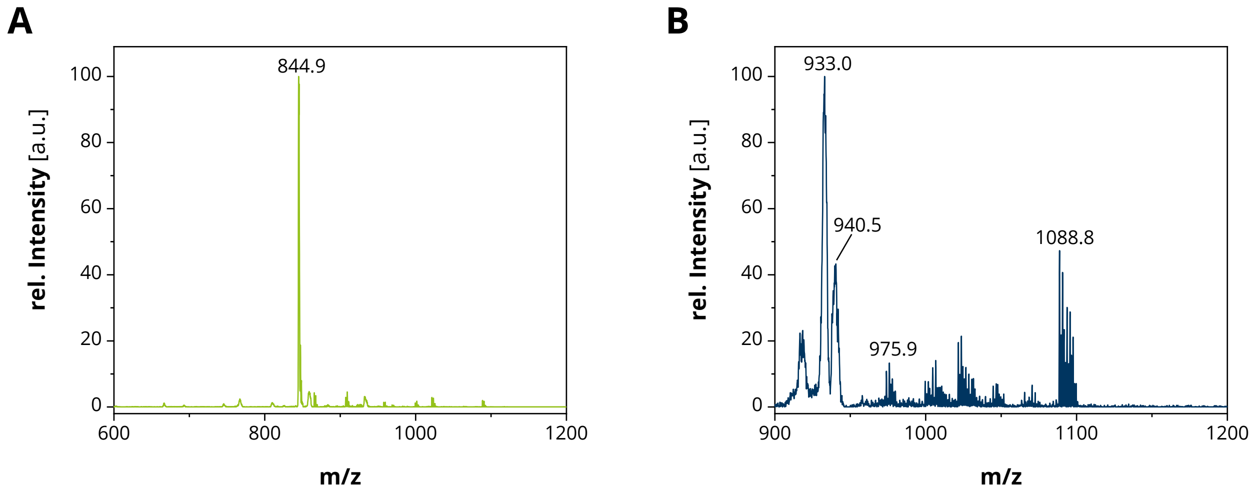

Figure S23 ESI-MS spectra of (A) **$Co^{II}Co^{II}$-{N^SN^N}$_m$** and (B) **$Zn^{II}Co^{II}$-{N^SN^N}$_m$**.

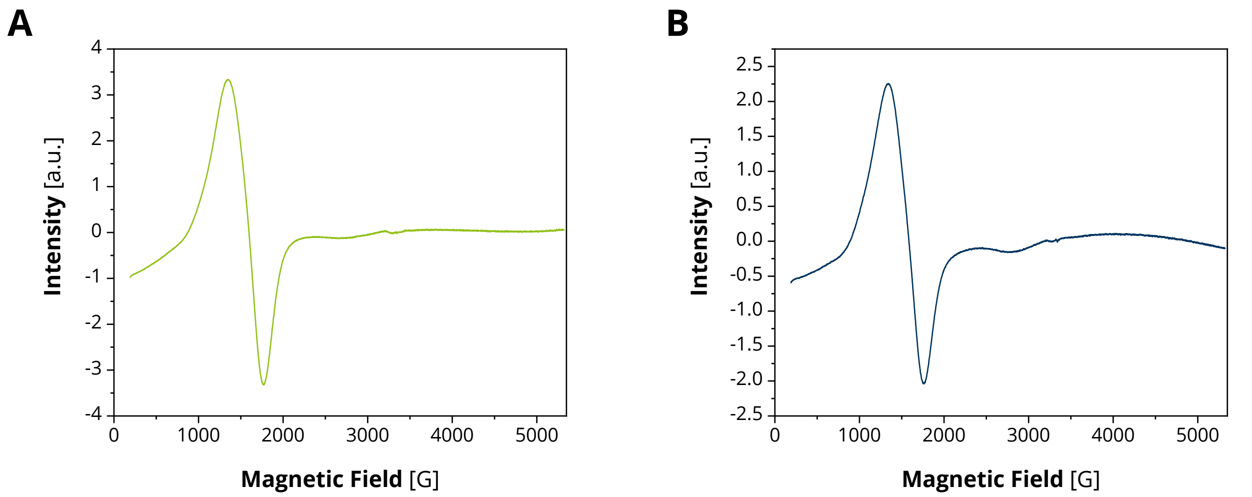

Figure S24 EPR spectra of (A) **$Co^{II}Co^{II}$-{N^SN^N}$_m$** and (B) **$Zn^{II}Co^{II}$-{N^SN^N}$_m$** in frozen MeCN (1 mM).

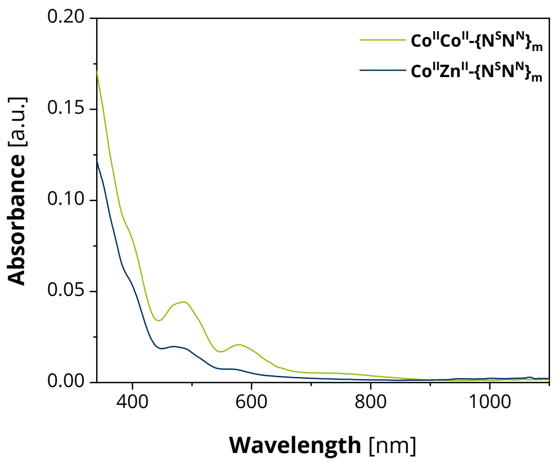

Figure S25 UV/vis/NIR spectra of **$Co^{II}Co^{II}$-{N^SN^N}$_m$** (green) and **$Zn^{II}Co^{II}$-{N^SN^N}$_m$** (blue) in MeCN (0.6 mM).

Table S4 Experimental data after 24 h photocatalysis with the standard procedure: *0.4 mM $[Ru(phen)_3](PF_6)_2$, 0.3 M TEOA, $MeCN/H_2O$ (4:1), CO_2, irradiation with blue LED light (λ = 450 nm, 1200 mcd, irradiation area 0.8 cm^2). The given values are averaged over three experiments with typical uncertainties of ±2-8%.

Entry	Catalyst	Deviation from standard procedure*	Conc. [µM]	Turnover frequency [min^{-1}]		Amount of product [µmol] (TON)		Selectivity CO [%]	Quantum yield [%]
				CO	H_2	CO	H_2		
1	**Cu^ICo^{II}-{N^SN^N}$_m$**	-	2	1.60	$3.28 \cdot 10^{-2}$	9.22 (2305)	0.189 (47.25)	98	0.15
2	**-**	-	-	0	$8.85 \cdot 10^{-3}$	0 (0)	0.0510 (12.75)	-	0
3	**Cu^ICo^{II}-{N^SN^N}$_m$**	w/o $[Ru(phen)_3](PF_6)_2$	2	0	$1.54 \cdot 10^{-3}$	0 (0)	0.00888 (2.220)	-	0
4	**Cu^ICo^{II}-{N^SN^N}$_m$**	w/o TEOA	2	0	$1.43 \cdot 10^{-3}$	0 (0)	0.00821 (2.053)	-	0
5	**Cu^ICo^{II}-{N^SN^N}$_m$**	w/o irradiation	2	0	0	0 (0)	0 (0)	-	0
6	**Cu^ICo^{II}-{N^SN^N}$_m$**	Ar instead CO_2	2	0	0	0 (0)	0 (0)	-	0.001
7	**Cu^ICo^{II}-{N^SN^N}$_m$**	MeCN	2	$3.84 \cdot 10^{-2}$	$8.44 \cdot 10^{-3}$	0.221 (55.25)	0.0485 (12.13)	82	0.004
8	**Cu^ICo^{II}-{N^SN^N}$_m$**	$MeCN/H_2O$ (9:1)	2	1.08	$4.34 \cdot 10^{-2}$	6.23 (1558)	0.250 (62.50)	96	0.10
9	**Cu^ICo^{II}-{N^SN^N}$_m$**	$MeCN/H_2O$ (1:1)	2	$1.37 \cdot 10^{-2}$	$3.85 \cdot 10^{-2}$	0.0791 (19.78)	0.0222 (5.550)	78	0.002
10	**Cu^ICo^{II}-{N^SN^N}$_m$**	+ 100 µL Hg^0	2	1.27	$3.72 \cdot 10^{-2}$	7.34 (1835)	0.214 (53.50)	97	0.12
11	**Cu^I-{N^SN^N}$_m$**	-	2	$8.06 \cdot 10^{-3}$	$4.84 \cdot 10^{-3}$	0.0464 (11.60)	0.0279 (6.975)	62	0.001
12	**Co^{II}-{N^SN^N}$_m$**	-	2	0.99	$2.34 \cdot 10^{-2}$	5.70 (1425)	0.135 (33.75)	98	0.09
13	**Cu^ICo^{II}-{N^SN^N}$_p$**	-	2	0.48	$1.17 \cdot 10^{-2}$	2.78 (695.0)	0.0671 (16.78)	98	0.04
14	**Cu^ICo^{II}-{N^SN^N}$_m$**	-	1.5	1.13	$1.97 \cdot 10^{-2}$	4.87 (1623)	0.0851 (28.37)	98	0.08
15	**Cu^ICo^{II}-{N^SN^N}$_m$**	-	1.0	1.09	$2.17 \cdot 10^{-2}$	3.13 (1565)	0.0626 (31.30)	98	0.05
16	**Cu^ICo^{II}-{N^SN^N}$_m$**	-	0.5	1.15	$2.92 \cdot 10^{-2}$	1.66 (1660)	0.0421 (42.10)	98	0.03
17	**$Co^{II}Co^{II}$-{N^NN^N}$_m$**	-	2	2.01	$6.51 \cdot 10^{-2}$	11.6 (2900)	0.375 (93.75)	97	0.19
18	**$Co^{II}Co^{II}$-{N^SN^N}$_m$**	-	2	1.11	$3.11 \cdot 10^{-2}$	6.39 (1598)	0.179 (44.75)	97	0.10
19	**$Zn^{II}Co^{II}$-{N^SN^N}$_m$**	-	2	1.08	$2.78 \cdot 10^{-2}$	6.21 (1553)	0.160 (40.00)	97	0.10
